# The adaptive evolution of cancer driver genes

**DOI:** 10.1186/s12864-023-09301-9

**Published:** 2023-04-25

**Authors:** Langyu Gu, Canwei Xia, Shiyu Yang, Guofen Yang

**Affiliations:** 1 State Key Laboratory for Biocontrol, School of Life Sciences, Sun Yat-sen University, Guangzhou, Guangdong 510275 China; 2 Ministry of Education Key Laboratory for Biodiversity and Ecological Engineering, College of Life Sciences, Beijing Normal University, Beijing, 100875 China; 3 The Affiliated Brain Hospital, Guangzhou Medical University, Guangzhou, 510180 Guangdong China; 4 Department of Gynecology, First Affiliated Hospital, Sun Yat-Sen University, Guangzhou, 510060 Guangdong China

**Keywords:** Cancer driver genes, Positive selection, Primates, Modern human populations, Thyroid cancer

## Abstract

**Background:**

Cancer is a life-threatening disease in humans; yet, cancer genes are frequently reported to be under positive selection. This suggests an evolutionary-genetic paradox in which cancer evolves as a secondary product of selection in human beings. However, systematic investigation of the evolution of cancer driver genes is sparse.

**Results:**

Using comparative genomics analysis, population genetics analysis and computational molecular evolutionary analysis, the evolution of 568 cancer driver genes of 66 cancer types were evaluated at two levels, selection on the early evolution of humans (long timescale selection in the human lineage during primate evolution, i.e., millions of years), and recent selection in modern human populations (~ 100,000 years). Results showed that eight cancer genes covering 11 cancer types were under positive selection in the human lineage (long timescale selection). And 35 cancer genes covering 47 cancer types were under positive selection in modern human populations (recent selection). Moreover, SNPs associated with thyroid cancer in three thyroid cancer driver genes (CUX1, HERC2 and RGPD3) were under positive selection in East Asian and European populations, consistent with the high incidence of thyroid cancer in these populations.

**Conclusions:**

These findings suggest that cancer can be evolved, in part, as a by-product of adaptive changes in humans. Different SNPs at the same locus can be under different selection pressures in different populations, and thus should be under consideration during precision medicine, especially for targeted medicine in specific populations.

**Supplementary Information:**

The online version contains supplementary material available at 10.1186/s12864-023-09301-9.

## Background

Cancer is one of the most life-threatening diseases with a high mortality rate in humans. Cancer gene mutations at the individual level are usually deleterious to the organism and negatively affect fitness [1, 2]. Thus, purifying selection of cancer gene mutations is necessary to maintain fitness. Surprisingly, studies have continuously found cancer genes to be under positive selection [3, 4], indicating their adaptive roles during evolution. Several hypotheses have been proposed to explain the evolutionary trade-off between positive selection and cancer risks, such as sexual selection, pathogen-host interactions, and genomic compensation [3, 5–8]. Studies have reported positively selected cancer genes in the ancestral lineage leading to human and other species [3, 7, 9], but few focused on positively selected cancer genes in the human lineage itself. The extent to which positive selection of cancer genes occurs along the human lineage during primate evolution remains unclear.

Notably, positive selection detection across the primate phylogeny usually detects selection in the earlier evolution of the human lineage on a large timescale, i.e., millions of years (*long timescale selection*) [10]. In contrast, detection at the population level reflects selection on a more recent time scale (*recent selection*) starting at the neolithic demographic transition and migration out of Africa in the last 100,000 years. During this period, modern humans have experienced significant changes in lifestyle and living environments [11, 12]. Diversified living environments and food resources, accompanied by new infectious diseases, represent new selective pressures on modern human populations [13]. These selective pressures work on new genotypes that are better adapted to novel environments. Thus, the last 100,000 years encompasses one of the most interesting time periods in human history [10, 14, 15]. A number of genes involved in local adaptations have been identified, such as genes involved in skin pigmentation [14], malaria response [16], hair morphology [17], and lactase persistence [18]. Genes related to human diseases have also been reported to be under recent positive selection due to their pleiotropic effects, such as genes related to neurodegenerative disease (HTT [19], APOE [20]). Although some cancer genes have also been reported to be under recent positive selection, such as the prostate cancer-related gene MLPH [21], systematic investigation of the role of recent selection on the adaptive evolution of cancer genes remains sparse.

Existing studies on the adaptive evolution of cancer genes have mainly focused on genes in specific cancer types or in limited species across the phylogeny [6, 7, 9, 22]. In addition, studies have not differentiated between selection of cancer driver genes and cancer passenger genes, which may be under different selection pressures [23]. With the rapidly developed next generation sequencing technologies and bioinformatics analysis, a recent study systematically analyzed more than 28,000 tumors of 66 cancer types and identified 568 cancer driver genes which is published in Nature Reviews Cancer [24]. In-depth evolutionary analyses of these cancer driver genes covering different cancer types across the phylogeny and in modern human populations may give us a more comprehensive picture of cancer gene evolution.

To obtain a deep understanding of cancer evolution, the adaptive evolution of 568 cancer driver genes in 66 different cancer types across the primate phylogeny (*long timescale selection*), as well as in modern human populations retrieved from the 1000 Genomes Project (*recent selection*), was evaluated. The main study aim was to determine the adaptive evolution of cancer driver genes at two levels, i.e. *selection on the early evolution* of *Homo sapiens* (detection across the primate phylogeny, i.e., millions of years timescale) and *recent selection* (detection among modern human populations, i.e., 100,000 years timescale). Findings from this study can have profound implications for understanding the evolution of cancer driver genes and provide clues for further precision medicine.

## Results

### Positive selection detection across the primate phylogeny

**Fig. 1:**
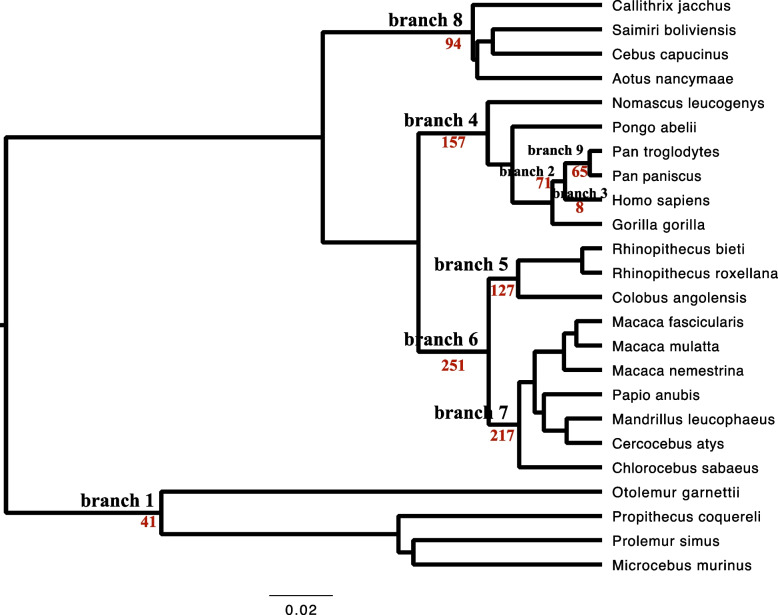
Cancer driver genes under positive selection under the branch-site model in PAML across the primate phylogeny. Target foreground branches were labeled. The number of genes under positive selection was given in red. The species tree was retrieved from the literature [37]. The unrooted tree was used for PAML analysis

The transcript IDs of 568 cancer driver genes were retrieved from the literature published in *Nature Reviews Cancer* [24]. To understand the evolutionary dynamics of these 568 cancer driver genes across the primate phylogeny, orthologous sequences in 23 primates were obtained using Oma inference [25]. Positive selection was detected in the main primate lineages under the branch-site model using PAML [26, 27]. Eight genes covering 11 cancer types were determined to be under positive selection in the human lineage under the branch-site model in PAML (Table 1), including the neuronal stress related gene, DROSHA [28], the immune related gene, LY75-CD302 [29], the neurodegenerative diseases related gene, RBFOX1 [30], the neurocognitive related gene, NRG1 [31], the viral and bacterial response related gene, STAT3 [32], the brain development related gene, NIN [33], the zinc finger protein gene, ZNF814, and the immune response and antiviral response related gene, TRAF3 [34]. Among which, three genes, including NIN, NRG1 and RBFOX1 have been reported under positive selection [33, 35, 36]. Compared to the human lineage, more genes were under positive selection in ancestral lineages leading to human and other primates (Fig. 1).

**Table 1:** The cancer driver genes under positive selection in the human lineage detected using PAML

Gene	Branch-site model				*P/2*	Sites under positive selection	Cancer types
	Model A	Model B	2ln(A-B)	ω		Uner BEB model with a posterior probability > 0.95	
DROSHA	-9569.2	-9593.43	48.44	202.65	< 0.01	142E, 150H, 1050 M, 1058E	LUSC,WT
LY75-CD302	-15,467	-15,535	136	200	< 0.01	1383Y,1388I,1393L,1394S,1402L,1412S,1413 V,	AML
						1417Q,1418G,1419 V,1423E,1428S,1429F,1430Q	
RBFOX1	-2118.24	-2147.37	58.27	270.69	< 0.01	297L,299Q,304A,305L,307P	PRAD
NRG1	-5366.86	-5379.37	25.02	94.63	< 0.01	2 K	ST
STAT3	-5563.45	-5577.78	28.65	92.15	< 0.01	N/A	DLBCL
NIN	-17,468.17	-17,477.49	18.64	791.16	< 0.01	N/A	BRCA,COREAD,MESO,ST
ZNF814	-4723.37	-4734.49	22.25	5.37	< 0.01	243H,268H	VV
TRAF3	-4022.31	-4033.59	22.56	999	< 0.01	N/A	MM

Model A: the alternative model in the branch-site test of positive selection in PAML

Model B: the null model in the branch-site test of positive selection in PAML

*ω*: the ratio of nonsynonymous (dN) to synonymous (dS) substitutions

*p*/2: the null distribution is a mixture distribution, and thus have to use *p* divided by 2 to test the hypothesis (PAML manual)

### Recent positive selection in modern human populations

Signals of recent positive selection were detected across the genome based on several methods. We used the haplotype-based test [38] first. Briefly, the frequency of selection-favored alleles can arise quickly, such that long-range association with the surrounding loci does not have time to be eliminated by recombination. The selected allele may thus be located at an unusually long haplotype with low diversity, which is in sharp contrast with the unselected genomic background. Therefore, in the haplotype-based method, various core haplotypes (haplotypes at a locus of interest) present at a single locus are used as internal controls to adjust for heterogeneity in the local recombination rate [15, 38], limiting the confounding effects of demography [39]. In addition, recently admixed populations and populations with close geographical proximity were excluded, similar to the strategy applied by the literature [10]. Since genome-wide empirical distributions were used for standardization in these haplotype-based tests, there was no formal significance test [38]. Two tests were applied, i.e. the integrated haplotype score (iHS) method and the cross-population extended haplotype homozygosity (xpEHH) method. The iHS method allows for discovery of more recent positively selected variants that have not reached fixation in the population [38], whereas the xpEHH method allows discovery of variants near/at fixation on long haplotypes in one population that remain polymorphic in other populations [14]. Under positive selection, SNPs with large absolute haplotype scores tended to be clustered together [38]. Therefore, to identify sites under positive selection, we counted the number of SNPs for which ∣iHS∣ or ∣xpEHH∣ > 2 in a 51-SNPs window. SNPs with ∣iHS∣ or ∣xpEHH∣ > 2 located at the top 1% 51-SNPs windows were considered under positive selection (thresholds of top 1% windows were given in the Supplementary File 1).

We also test for differences in allele frequencies between populations (*F*_*ST*_) [40]. Since *F*_*ST*_ and allele frequency are highly correlated, to define a high *F*_*ST*_, we grouped *F*_*ST*_ values in different allele frequency bins and contrasted them to their own frequency classes. The top 5% *F*_*ST*_ values in its own frequency bin were statistically significally high. Therefore, we applied different *F*_*ST*_ thresholds to identify positively selected sites for alleles falling into different frequency bins (Supplementary File 2). SNPs under positive selection detected by both haplotype-based tests (iHS or xpEHH) mentioned above, and meanwhile with high *F*_*ST*_ values were considered under recent positive selection [14].

The haplotype-based tests have already limited the confounding effects of demography as we mentioned above. We also excluded recent admixed populations and populations with close geographical proximity to further minimize demographic effects. To make our results more convinced, we further applied the population branch statistics (PBS) test to account for demography. PBS value represents the amount of allele frequency change at a given locus in a population since its divergence from the other two populations [41]. SNPs with statistically significantly high PBS values (top 5%) were considered under positive selection (PBS thresholds can be found in the Supplementary File 3).

**Fig. 2:**
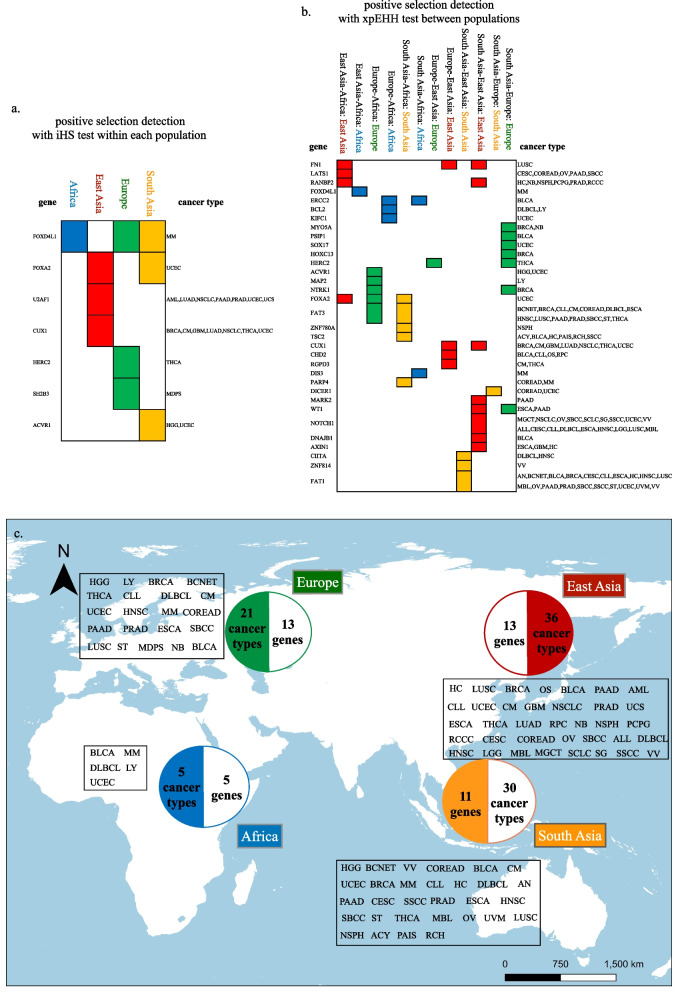
Recent positive selection detection in modern human populations. **a** Positive selection detection using the iHS test within each population. Positively selected cancer driver genes and corresponding cancer types were given. **b** Positive selection detection using the xpEHH test between populations. Positively selected cancer driver genes and corresponding cancer types were given. **c** Corresponding cancer types of positively selected cancer driver genes in different populations. Abbreviations of cancer types can be found in the Supplementary File 6

Because the main goal of this study was to explore the evolution of coding regions, only those SNPs located in exons under positive selection were thus chosen for further study. Seven genes across 14 cancer types were identified under positive selection using the iHS test and the *F*_*ST*_ test (Fig. 2a). One pigmentation related gene, HERC2, has been reported under positive selection before [42, 43]. Gene SH2B3 which functions in anti-bacteria defense also has been reported under positive selection [44]. Using the xpEHH test and the *F*_*ST*_ test, 33 genes across 44 cancer types were identified under positive selection (Fig. 2b). Three genes had been previously reported to be under positive selection, including the gene HERC2 mentioned above, the psychiatric disorder-relevant gene, FAT1 [45], and the human skull shape and morphology determination-related gene, RGPD3 [46]. Most positively selected genes we identified here are related to immune and neurodevelopment (Supplementary File 4). The site frequency spectrum of each locus under positive selection was plotted (Supplementary File 5). Abbreviations of cancer types can be found in the Supplementary File 6.

PBS tests showed that except three SNPs of FN1, one SNP of CHD2, one SNP of HERC2, one SNP of CIITA, one SNP of ZNF814, one SNP of SOX17, one SNP of PSIP1, and one SNP of DICER1 (highlighted in the Supplementary File 7), other positively selected SNPs detected based on the haplotype-based methods (iHS and xpEHH) and the *F*_*ST*_ method were all with significantly high PBS values. And this only affectted six genes (CHD2, CIITA, ZNF814, SOX17, PSIP1 and DICER1) in the final positively selected gene list. Therefore, signals we identified with the haplotype-based methods (iHS and xpEHH) and the *F*_*ST*_ test were very likely due to positive selection instead of demography. Since the PBS test cannot detect positively selected signals in the African population (because always as the outgroup), we chose positively selected signals identified based on both the haplotype-based tests as well as the *F*_*ST*_ test, but also presented corresponding PBS values (Supplementary File 7).

Cancer driver genes under positive selection exhibited population-specific patterns (Fig. 2, Supplementary File 7). There were 13 genes (CUX1, U2AF1, FOXA2, RANBP2, LATS1, FN1, CHD2, RGPD3, DNAJB1, AXIN1, NOTCH1, WT1 and MARK2) corresponded to 36 cancer types under positive selection in East Asia; five genes (FOXD4L1, ERCC2, BCL2, KIFC1 and DIS3) corresponded to five cancer types under positive selection in Africa; 13 genes (HERC2, FOXD4L1, SH2B3, ACVR1, MAP2, FOXA2, NTRK1, FAT3, HOXC13, SOX17, WT1, PSIP1 and MYO5A) corresponded to 21 cancer types under positive selection in Europe; and 11 genes (FOXA2, FOXD4L1, ACVR1, TSC2, PARP4, ZNF780A, FAT3, CIITA, ZNF814, FAT1 and DICER1) corresponded to 30 cancer types under positive selection in South Asia.

## THCA-associated SNPs under positive selection

**Fig. 3:**
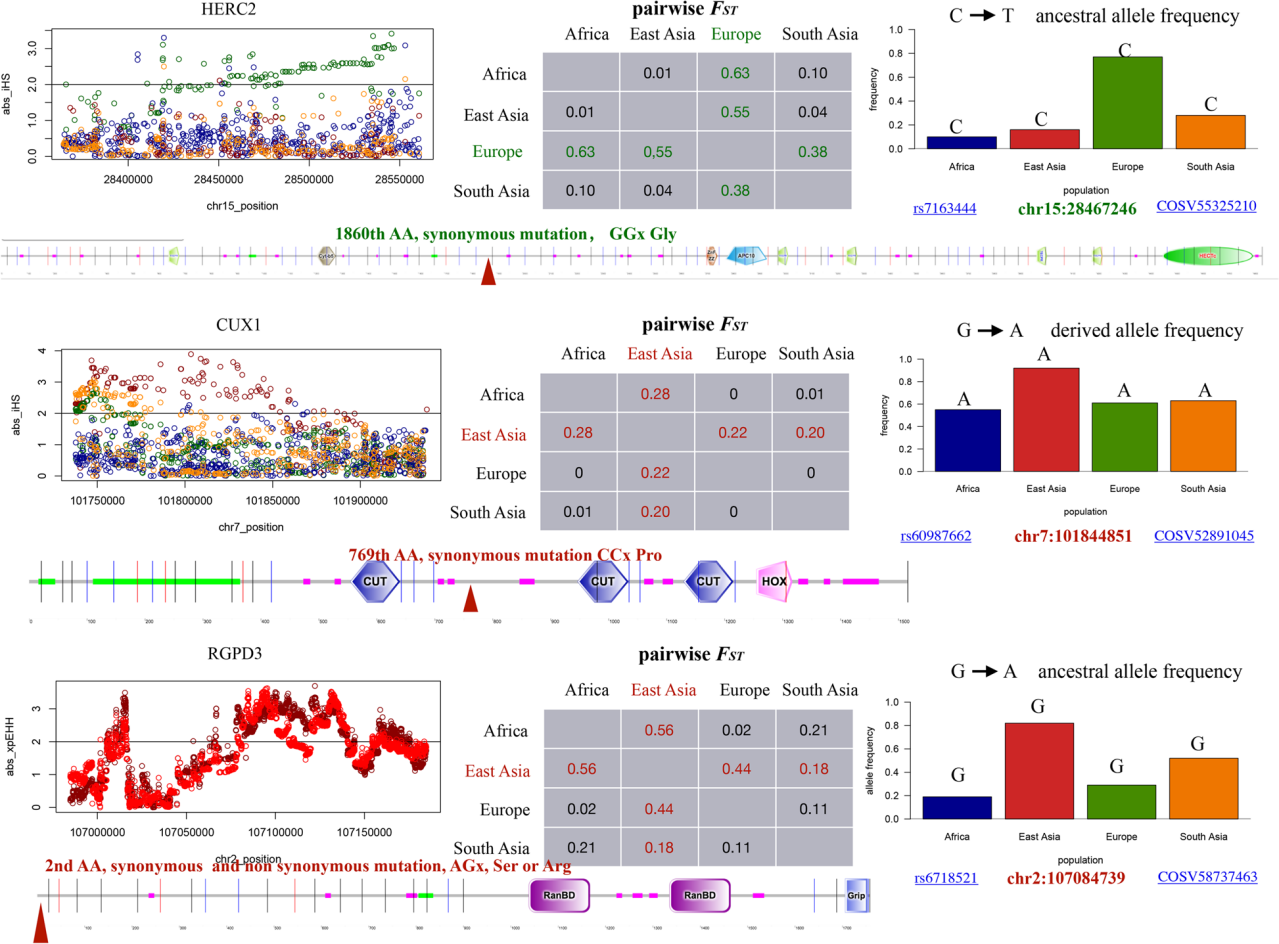
Thyroid cancer (THCA) associated SNPs of three THCA driver genes under positive selection in the East Asian population and the European population. High iHS or xpEHH(> 2) scores plottings, pairwise *F*_*ST*_ comparisons, allele frequencies, and positions of SNPs on the corresponding secondary protein structure were given. THCA-associated SNP of the driver gene HERC2 showed positive selection signals in the European population, and the THCA-associate SNPs of driver genes CUX1 and RGPD3 showed positive selection signals in the East Asian population. SNP IDs in Ensembl and COSMIC databases were given

To see whether sites under positive selection happen to be cancer-associated sites, we screened all positively selected SNPs we identified here in the COSMIC database, the largest and most comprehensive resources of somatic mutations in human cancer [47]. Interestingly, SNPs associated with thyroid cancer (THCA) in three THCA driver genes were found to be under positive selection in the East Asian population (CUX1 and RGPD3) and the European population (HERC2) (Fig. 3). The locus chr7:101844851 of the CUX1 gene was located within clusters showing an ∣iHS∣ > 2 and high pairwise *F*_*ST*_ values between the East Asian and other populations. Allele frequencies were consistent with these results, i.e., there was a higher frequency of derived SNP that was positively selected in the East Asian population. CUX1 belongs to the homeodomain transcription factor family, and is involved in various physiological events, including tissue developement and tumorigenesis [48], but has not been previously reported to be under positive selection. Similarly, the locus chr15:28467246 of the HERC2 gene was located within clusters showing an ∣iHS∣ > 2 and high pairwise *F*_*ST*_ value between the European and other populations (Fig. 3). Allele frequencies were consistent with these results, i.e. a higher frequency of the positively selected ancestral SNPs was present in the European population. HERC2 has been reported to be under positive selection and is related to pigmentation [43]. Mutations in the SNPs of these two genes are synonymous and are not located on any known protein domains (Fig. 3). A cross-population test using xpEHH found the the locus chr2:107084739 of the RGPD3 gene, the human skull shape and face morphology determination-related gene [46], was located within clusters with an ∣xpEHH∣ > 2 in the East Asian population when compared to both the European and South Asian populations. Mutation of this SNP in RGPD3 can be synonymous or nonsynonymous (Ser or Arg), and is also not located on any known protein domains (Fig. 3). Thus, THCA-associated SNPs under positive selection were identified in populations of East Asia and Europe.

### THCA incidence in different populations

**Fig. 4:**
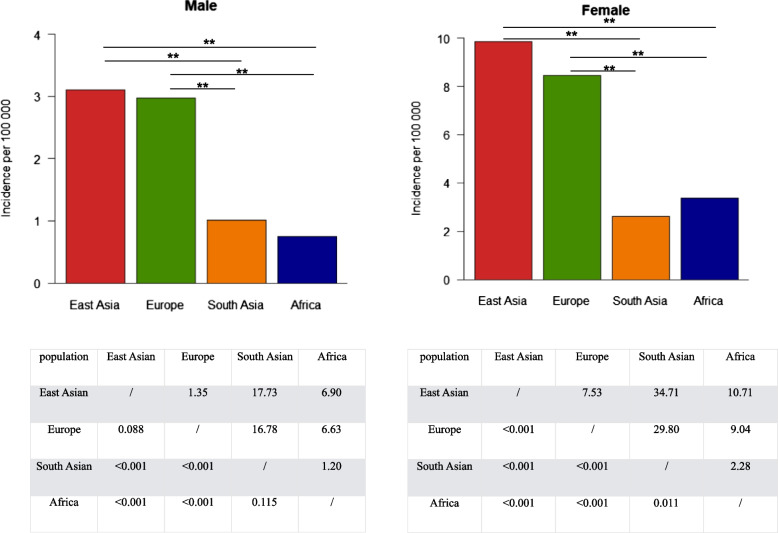
Thyroid cancer (THCA) incidence comparisons among different populations by Z test. The incidence were significantly higher in both the East Asian population and the European population than other populations. Z statistics were given in the upper triangle, while *p* values were given in the lower triangle

Identification of THCA-associated SNPs under positive selection in East Asian and European populations could lead to higher incidence of THCA in these populations. To determine whether this is the case, global THCA incidence was evaluated. THCA incidence varied among populations (Fig. 4, Supplementary File 8). For males, there were high incidences in the East Asian poppulation (3.11 per 100 000 persons) and in the European population (2.97 per 100 000 persons), and relative low incidences in the South Asian population (1.01 per 100 000 persons) and the African population (0.75 per 100 000 persons). The pattern in females was similar. For females, there were high incidences in the East Asian population (9.85 per 100 000 persons) and the European population (8.44 per 100 000 persons), and relative low incidences in the African population (3.36 per 100 000 persons) and the South Asian population (2.61 per 100 000 persons). The THCA incidences were significantly different between high incidence group (East Asia and Europe) and low group (Africa and South Asia) in both males and females.

## Discussion

A few studies have already conducted a molecular evolutionary analysis of cancer genes across primates and found many cancer genes were under positive selection in primates [7]. We also found hundreds of genes under positive selection in nonhuman primates (Fig. 1). This indicates that many cancer driver genes play adaptive roles during the early evolution of primates. The genetic background of many cancer genes can be traced back long before the human lineage. For example, some cancer driver genes are essential biological genes controlling basic biology, such as DNA damage repair, cell division [49–51]. As we mentioned in the Introduction section, several hypotheses have already been proposed to explain this phenomenon. For example, viruses can exert a powerful selective pressure and be the key driver of adaptive mutations in proteins [8], which has already been demonstrated in cancer driver genes [7].

However, when focusing on positive selection in the human lineage itself, only eight cancer driver genes were under positive selection (Table 1, Fig. 1). This may be because many cancer driver genes served adaptive roles in the early evolution of primates, but did not work in the human lineage. Massive changes occurred during the evolution of human beings, such as upright walking [52–54] and brain development [55], which can induce different selection pressures in the evolution of the human lineage. Indeed, most genes under positive selection in the human lineage we identified here are related to neurocognitive, brain development and immune responses. In addition, nonhuman primates may possess alternative cancer genetic bases or anticancer mechanisms, as these are not rare in mammals and can be seen in mole rats, elephants, and whales [56].

In modern human populations, there were 35 cancer driver genes covering 47 cancer types under recent positive selection. Genetic disease may occur as a by-product of evolution due to genetic pleiotropic effects. For instance, the seemingly human-specific disease of schizophrenia and the greater human susceptibility to Alzheimer’s disease may be a by-product of human specialization for higher cognitive function [36, 57]. Significant changes in modern human populations but not in long timescale evolution are also found in the evolution of testis-related genes [10]. Similar alterations may be observed in human cancers. Identification of cancer driver genes under recent positive selection supports adaptive roles for these genes and may help survival of these cancer driver genes and accumulation of cancer-related mutations during evolution.

We further found THCA-associated SNPs in three THCA driver genes, CUX1, HERC2 and RGPD3, were determined to be under positive selection in East Asian and European populations. SNP mutations of CUX1 and HERC2 are synonymous mutations. Studies have already shown that synonymous SNPs under positive selection are pervasive in mammals; for example, they affect mRNA levels [58] and translation via mRNA destabilization [59] or create internal promoter sites [60]. Synonymous mutations frequently function as driver mutations in human cancers [61] and can be under positive selection [62]. Functions of THCA-associated SNPs under positive selection we identified here requires further study. We also found a higher incidence of THCA in East Asian and European populations compared to other populations. Both environmental conditions [63] and genetic components [64] are important factors to affect cancer occurence. Although we cannot exclude other effects on the high incidence rates in East Asian and European populations, the THCA-associated SNPs under positive selection we identified here at least provides one genetic clue to explain the high incidence of THCA in corresponding populations.

## Conclusions

Cancer can be evolved, in part, as a by-product of adaptive changes in humans. Different SNPs at the same locus can be under different selection pressures in different populations, and thus should be under consideration during precision medicine, especially targeted medicine in specific populations.

## Methods

### Orthologous sequences inference

The transcript IDs of 568 cancer driver genes were retrieved from the literature published in *Nature Reviews Cancer* [24] (Supplementary File 9). To understand the evolutionary dynamics of these 568 cancer driver genes across the primate phylogeny, orthologous sequences of 23 primates were retrieved from Ensembl Biomart (https://www.ensembl.org/index.html). Oma was used to perform further orthologous sequence inference with default parameters [25]. The species tree retrieved from the literature [37] was used for orthologous sequences inference using oma, with *Mus musculus* as the outgroup.

### Positive selection detection across the phylogeny of primates

Positive selection detection was based on the rates of protein evolution calculated using the branch-site model with codeml in PAML [26, 27]. The ratio of nonsynonymous (dN) to synonymous (dS) substitutions (*ω*) can vary over sites and time. We thus used the branch-site model which considers the variation in *ω* both among sites and across branches to detect positive selection affecting sites along the target lineage (foreground branch). If a nonsynonymous mutation is less likely to be tolerated during evolution, *ω* will be <  < 1, meaning that purifying selection takes effects. If there is no selection pressure (neutral selection), *ω* will be equal to 1. If changes of nonsynonymous mutations have beneficial effects and are favored by selection, *ω* will be >  > 1, indicating that they are under positive selection. Multiple sequence alignment for codon alignment was conducted using MAFFT within T-coffee [65] using default parameters. An unrooted primate phylogenetic tree retrieved from the literature [37] was used for the calculation (Fig. 1).

Nine branches representing the main primate lineages were set as foreground branches to run the branch-site model separately (Fig. 1). Parameter settings and test for statistical significance were the same as those previously published [66]. Briefly, initial branch lengths were estimated using the M0 model, and fixed branch lengths (fix_blength = 2) were used for downstream analysis. We removed alignment gaps and ambiguity characters by setting Cleandata = 1. Comparisons were made between the modified ModelA (model = 2, NSsites = 2) and the ModelB (a null model with *ω*2 = 1 fixed (fix_omega = 1 and omega = 1)). A likelihood ratio test (LRT) was then used to calculate a *chi*-square approximation, and the *p*/2 value was used to consider mixture distribution [26, 27]. Bonferroni correction was used for multiple test corrections [67]. Sites under positive selection were identified under BEB (Bayes empirical Bayes) with a posterior probability > 0.95 [26, 27].

### Recent positive selection detection in modern human populations

Phased genetic data of the 1000 Genomes Project of modern human populations were retrieved from ftp.1000genomes.ebi.ac.uk/vol1/ftp/release/20130502. The populations used were the same as those in the recently published literature [10], which excluded recently admixed populations or populations in close geographic proximity. Details of these populations can be found in Supplementary File 10. 1) Populations of African ancestry (*n* = 311) included Gambians in Western Division, The Gambia (GWD, *n* = 113), Luhya in Webuye, Kenya (LWK, *n* = 99) and Esan in Nigeria (ESN, *n* = 99); 2) Populations of East Aisan ancestry (*n* = 306) included Han Chinese in Bejing, China (CHB, n = 103), Japanese in Tokyo, Japan (JPT, *n* = 104), and Kinh in Ho Chi Minh City, Vietnam (KHV, *n* = 99); 3) Populations of European ancestry (*n* = 297) included British people in England and Scotland, United Kingdom (GBR, *n* = 91), Finnish people in Finland (FIN, *n* = 99), and Toscani people in Italy (TSI, *n* = 107)); and 4) Populations of South Asian ancestry (*n* = 284) included Bengali people in Bangladesh (BEB, *n* = 86), Indian Telugu people in the United Kingdom (ITU, *n* = 102), and Punjabi people in Lahore, Pakistan (PJL, *n* = 96).

PLINK and VCFtools were used to process variant call format (VCF) files for autochromosomes [68]. SNPs with indels were removed. The GRCh37/hg19 reference genome from Ensembl was used to retrieve genomic location and identify SNPs in coding sequences of cancer driver genes here. The iHS and xpEHH methods, implemented in selscan were used to detect genome-wide positive selection using default parameters [69]. Biallelic SNPs with minor allele frequency (MAF) ≥ 0.05 were considered for positive selection detection. Ancestral allelels have already been annotated in the vcf files [70]. Unstandardized iHS and xpEHH scores were normalized in frequency bins across the entire genome using the script *norm* in the selscan program. Pairwise *F*_*ST*_ was calculated using the Weir & Cockerham *F*_*ST*_ calculation in VCFtools. Allele frequencies in each population were calculated using VCFtools with parameters –keep and –frequency. The VCF files were indexed and target regions were extracted with tabix [71]. Then the site frequency spectrums of each locus under positive selection were plotted for different popoulations.

Details about PBS tests were described in the literature [41]. Briefly, the *F*_*ST*_ for each SNP was first log-transformed [72]:

$$T=-log\left(1-{F}_{ST}\right)$$

Then the PBS of test population since the divergent from the sister population was obtained as:

$$PBS=\left({T}^{test-sister}+{T}^{test-Africa}-{T}^{sister-Africa}\right)/2$$

We thus calculated PBS values across the genome as follows, with the African population as the outgroup.

- 1) the European population diverged from the East Asian population;
  $${PBS}^{1}={T}^{Europe-EastAsia}+{T}^{Europe-Africa}-{T}^{EastAsia-Africa}/2$$
- 2) the European population diverged from the South Asian population;
  $${PBS}^{2}={T}^{Europe-SouthAsia}+{T}^{Europe-Africa}-{T}^{SouthAsia-Africa}/2$$
- 3) the East Asian population diverged from the South Asian population;
  $${PBS}^{3}={T}^{EastAsia-SouthAsia}+{T}^{EastAsia-Africa}-{T}^{SouthAsia-Africa}/2$$
- 4) the East Asian population diverged from the European population;
  $${PBS}^{4}={T}^{EastAsia-Europe}+{T}^{EastAsia-Africa}-{T}^{Europe-Africa}/2$$
- 5) the South Asian population diverged from the European population;
  $${PBS}^{5}={T}^{SouthAsia-Europe}+{T}^{SouthAsia-Africa}-{T}^{Europe-Africa}/2$$
- 6) the South Asian population diverged from the East Asian population.
  $${PBS}^{6}={T}^{SouthAsia-EastAsia}+{T}^{SouthAsia-Africa}-{T}^{EastAsia-Africa}/2$$

We then plotted the distribution of PBS values across the genome in each comparison and top 5% PBS values were considered as statistically significantly high. Since we have to use the African population as the outgroup, we thus cannot calculate PBS values for African population-related comparisons.

### Protein domain prediction

Protein domains were predicted using the Simple Modular Architecture Research Tool (SMART) with default parameters [73].

### Cancer incidence comparisons

THCA data were retrieved from the Cancer Incidence in Five Continents Vol. XI [74]. To accompany the population genetic analysis above, the same populations or nearby populations were used in cancer incidence rate analysis. The age-standardized rates (ASR) were used for comparisons. In Africa, the THCA incidence ranges from 0 to 1 per 100 000 males, and 1.9 to 4.4 per 100 000 females from 3 registries involving 5 465 978 persons; in East Asia, the THCA incidence ranges from 0.3 to 7.6 per 100 000 males, and 0.5 to 22 per 100 000 females from 45 registries involving 107 646 738 persons; in Europe, the THCA incidence ranges from 1.5 to 10.6 per 100 000 males, and 3.3 to 38.5 per 100 000 females from 41 registries involving 101 816 542 persons; in South Asia, the THCA incidence ranges from 0.1 to 3.8 per 100 000 males, and 0.8 to 9.7 per 100 000 females from 16 registries involving 50 983 915 persons; Z test was used to compare the THCA incidence among different populations (Africa, East Asia, South Asian, Europe) in males and females, respectively. The statistic Z is calculated by $\frac{\left|\frac{{\mathrm{c}}_{1}}{{\mathrm{n}}_{1}}-\frac{{\mathrm{c}}_{2}}{{\mathrm{n}}_{2}}\right|}{\sqrt{(\frac{{\mathrm{c}}_{1}+{\mathrm{c}}_{2}}{{\mathrm{n}}_{1}+{\mathrm{n}}_{2}})*\left(1-\frac{{\mathrm{c}}_{1}+{\mathrm{c}}_{2}}{{\mathrm{n}}_{1}+{\mathrm{n}}_{2}}\right)*(\frac{1}{{\mathrm{n}}_{1}}+\frac{1}{{\mathrm{n}}_{2}})}}$, where ${\mathrm{c}}_{1}$ and ${\mathrm{c}}_{2}$ represent the number of THCA patients in population 1 and population 2, respectively; ${\mathrm{n}}_{1}$ and ${\mathrm{n}}_{2}$ represent the population size in population 1 and population 2, respectively.

## Supplementary Information

**Additional file 1. **

**Additional file 2. **

**Additional file 3. **

**Additional file 4. **

**Additional file 5. **

**Additional file 6. **

**Additional file 7. **

**Additional file 8. **

**Additional file 9. **

**Additional file 10. **

## Data Availability

All data generated or analysed during this study are included in this published article and its supplementary information files. Five hundred sixty-eight cancer driver genes we analyzed were retrieved from [24] (Supplementary File 9). Homologous sequences were retrieved from Ensembl database https://asia.ensembl.org/index.html. Phased genetic data of the 1000 Genomes Project of modern human populations were retrieved from ftp.1000genomes.ebi.ac.uk/vol1/ftp/release/20130502/.

## Abbreviations

dN: Nonsynonymous
dS: Synonymous
BEB: Bayes empirical Bayes
iHS: The integrated haplotype score
xpEHH: The cross-population extended haplotype homozygosity
PBS: Population branch statistics
THCA: Thyroid cancer
LRT: Likelihood ratio test
GWD: Gambians in Western Division, The Gambia
LWK: Luhya in Webuye, Kenya
ESN: Esan in Nigeria
CHB: Han Chinese in Bejing, China
JPT: Japanese in Tokyo, Japan
KHV: Kinh in Ho Chi Minh City, Vietnam
GBR: British people in England and Scotland, United Kingdom
FIN: Finnish people in Finland
TSI: Toscani people in Italy
BEB: Bengali people in Bangladesh
ITU: Indian Telugu people in the United Kingdom
PJL: Punjabi people in Lahore, Pakistan
VCF: Variant call format
MAF: Minor allele frequency
SMART: The Simple Modular Architecture Research Tool
ASR: Age-standardized rates
