# Supplementary material for: The adaptive evolution of cancer driver genes
Source: BMC Genomics. 2023 Apr 25;24:215. doi: 10.1186/s12864-023-09301-9 (PMC10131384; doi:10.1186/s12864-023-09301-9)
Supplement: Supplementary file 1 — Additional file 1. [file 12864_2023_9301_MOESM1_ESM.pdf]

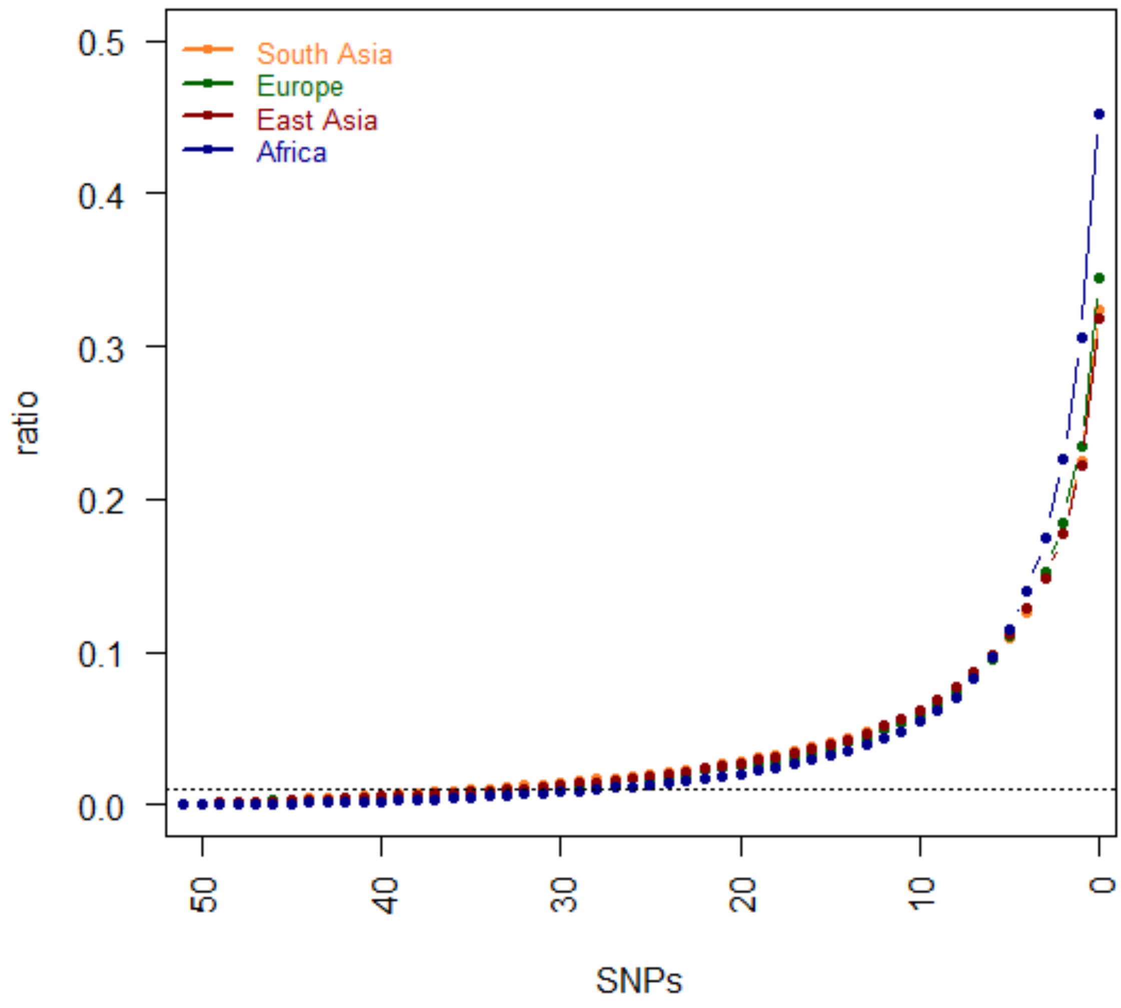

Figure S1 The distribution of the number of SNPs with large │iHS│ values in a 51-SNPs window across the genome.X-axis represents the number of SNPs with │iHS│>2 in a 51-SNPs window. Y-axis represents top ratio across the genome. Top 1% was set as the threshold.

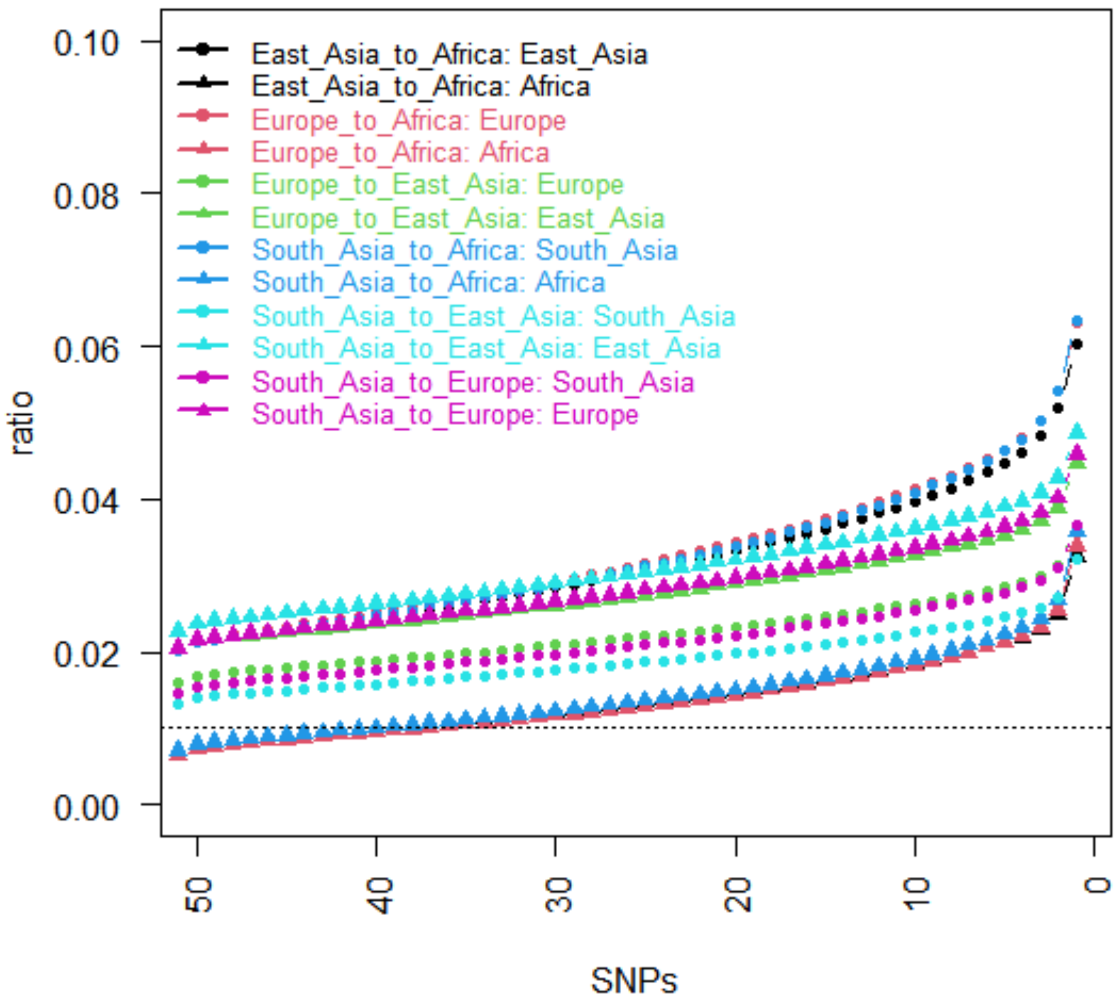

Figure S2 The distribution of the number of SNPs with large │xpEHH│ values in a 51-SNPs window across the genome. X-axis represents the number of SNPs with │xpEHH│>2 in a 51-SNPs window. Y-axis represents top ratio across the genome. Top 1% was set as the threshold.
