# Supplementary material for: The adaptive evolution of cancer driver genes
Source: BMC Genomics. 2023 Apr 25;24:215. doi: 10.1186/s12864-023-09301-9 (PMC10131384; doi:10.1186/s12864-023-09301-9)
Supplement: Supplementary file 2 — Additional file 2. [file 12864_2023_9301_MOESM2_ESM.pdf]

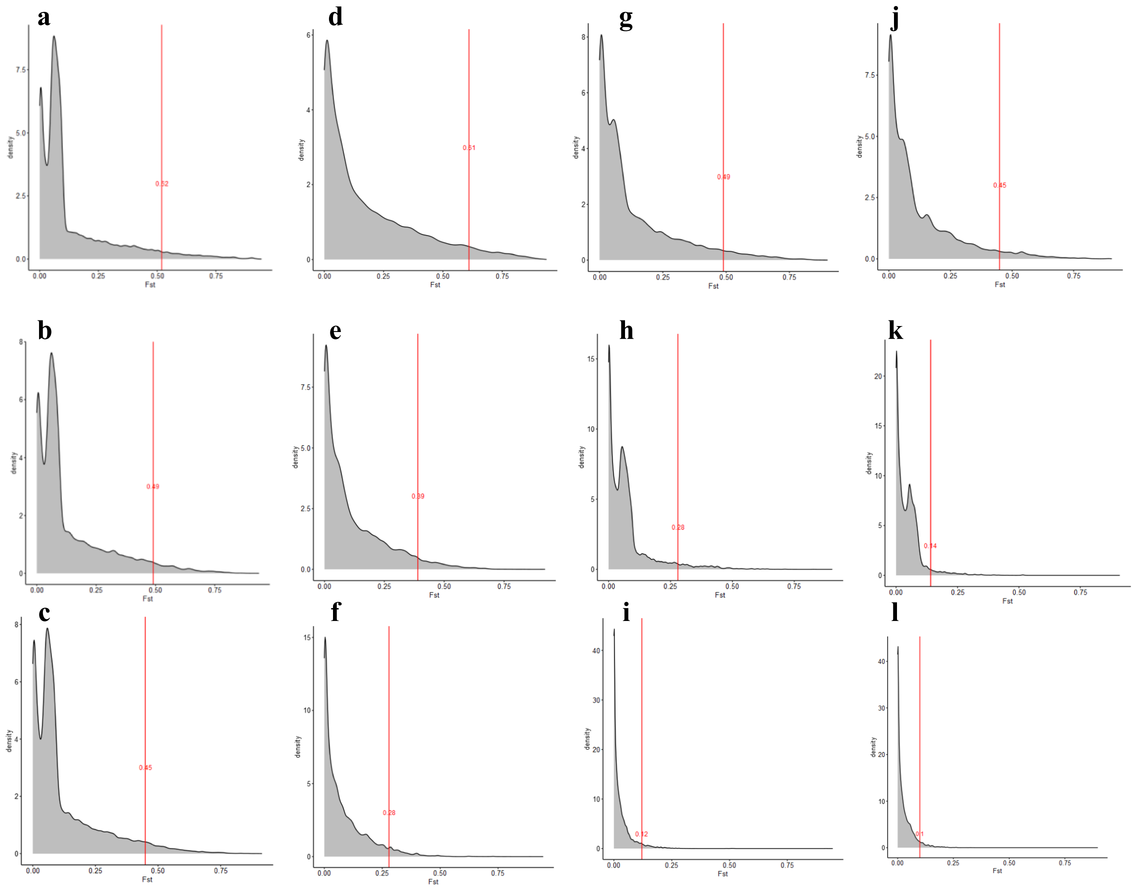

Figure S1 Pairwise F_{ST} distributions. The top 5% thresholds were given. (a) comparisons between the African population and the East Asian population for allele 1 falling into the allele frequency bin between 0.05-0.10 in the African population; (b) comparisons between the African population and the European population for allele 1 falling into the allele frequency bin between 0.05-0.10 in the African population; (c) comparisons between the African population and the South Asian population for allele 1 falling into the allele frequency bin between 0.05-0.10 in the African population; (d) comparisons between the East Asian population and the African population for allele 1 falling into the allele frequency bin between 0.05-0.10 in the East Asian population; (e) comparisons between the East Asian population and the European population for allele 1 falling into the allele frequency bin between 0.05-0.10 in the East Asian population; (f) comparisons between the East Asian population and the South Asian population for allele 1 falling into the allele frequency bin between 0.05-0.10 in the East Asian population; (g) comparisons between the European population and the African population for allele 1 falling into the allele frequency bin between 0.05-0.10 in the European population; (h) comparisons between the European population and the East Asian population for allele 1 falling into the allele frequency bin between 0.05-0.10 in the European population; (i) comparisons between the European population and the South Asian population for allele 1 falling into the allele frequency bin between 0.05-0.10 in the European population; (j) comparisons between the South Asian population and the African population for allele 1 falling into the allele frequency bin between 0.05-0.10 in the South Asian population; (k) comparisons between the South Asian population and the East Asian population for allele 1 falling into the allele frequency bin between 0.05-0.10 in the South Asian population; (l) comparisons between the South Asian population and the European population for allele 1 falling into the allele frequency bin between 0.05-0.10 in the South Asian population.

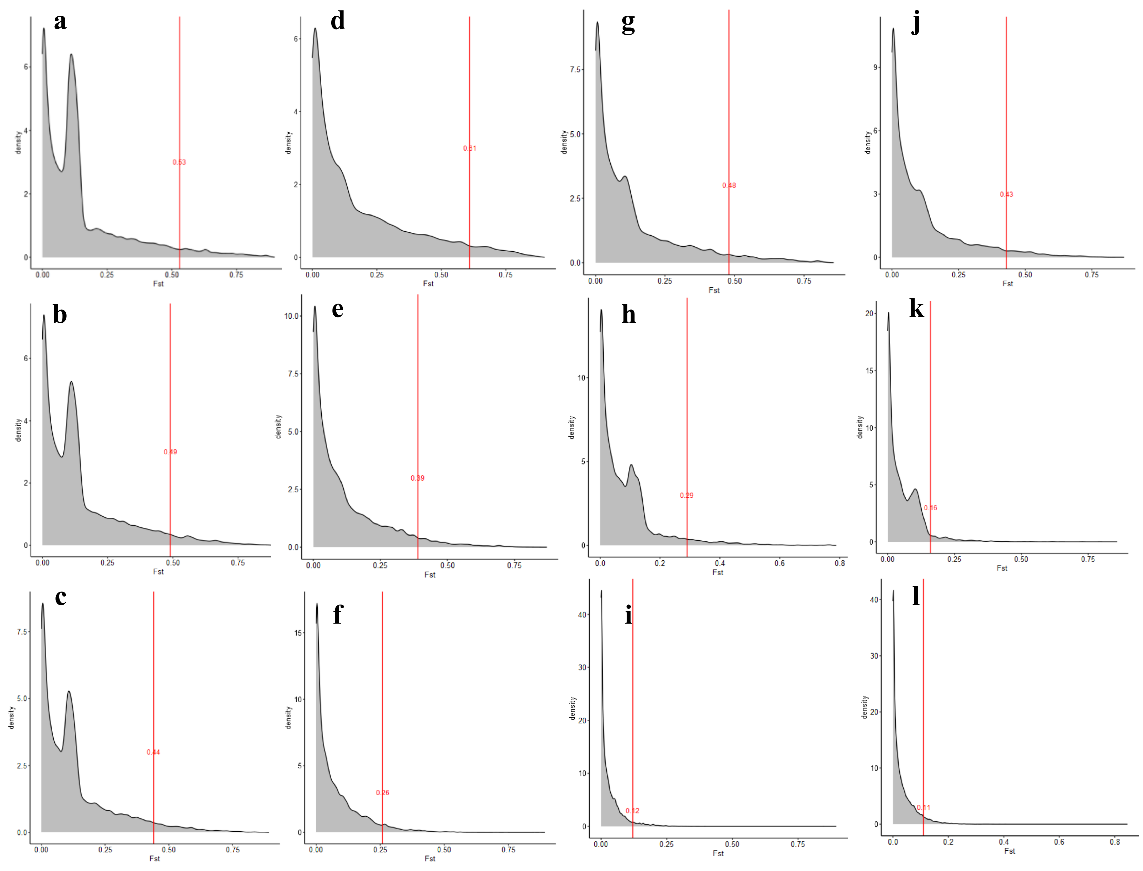

Figure S2 Pairwise F_{ST} distributions. The top 5% thresholds were given. (a) comparisons between the African population and the East Asian population for allele 1 falling into the allele frequency bin between 0.10-0.15 in the African population; (b) comparisons between the African population and the European population for allele 1 falling into the allele frequency bin between 0.10-0.15 in the African population; (c) comparisons between the African population and the South Asian population for allele 1 falling into the allele frequency bin between 0.10-0.15 in the African population; (d) comparisons between the East Asian population and the African population for allele 1 falling into the allele frequency bin between 0.10-0.15 in the East Asian population; (e) comparisons between the East Asian population and the European population for allele 1 falling into the allele frequency bin between 0.10-0.15 in the East Asian population; (f) comparisons between the East Asian population and the South Asian population for allele 1 falling into the allele frequency bin between 0.10-0.15 in the East Asian population; (g) comparisons between the European population and the African population for allele 1 falling into the allele frequency bin between 0.10-0.15 in the European population; (h) comparisons between the European population and the East Asian population for allele 1 falling into the allele frequency bin between 0.10-0.15 in the European population; (i) comparisons between the European population and the South Asian population for allele 1 falling into the allele frequency bin between 0.10-0.15 in the European population; (j) comparisons between the South Asian population and the African population for allele 1 falling into the allele frequency bin between 0.10-0.15 in the South Asian population; (k) comparisons between the South Asian population and the East Asian population for allele 1 falling into the allele frequency bin between 0.10-0.15 in the South Asian population; (l) comparisons between the South Asian population and the European population for allele 1 falling into the allele frequency bin between 0.10-0.15 in the South Asian population.

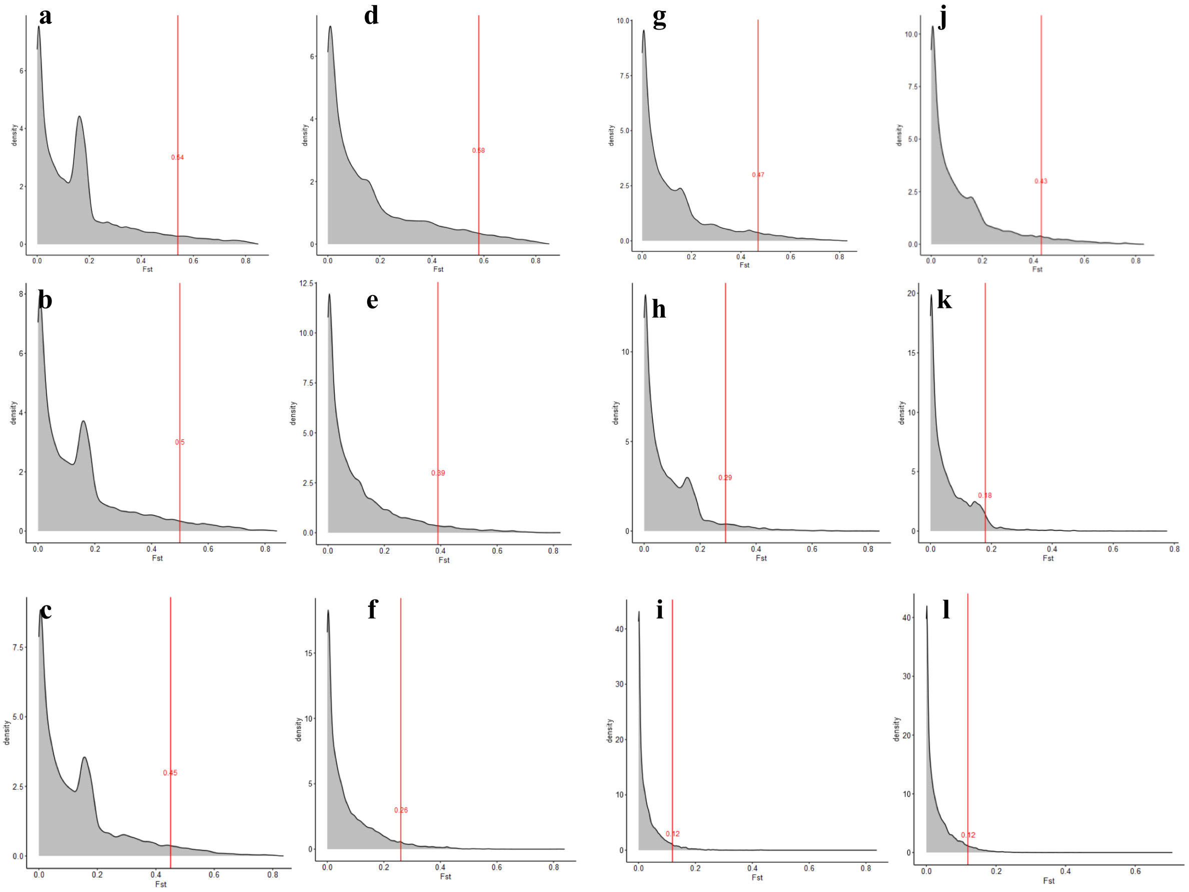

Figure S3 Pairwise F_{ST} distributions. The top 5% thresholds were given. (a) comparisons between the African population and the East Asian population for allele 1 falling into the allele frequency bin between 0.15-0.20 in the African population; (b) comparisons between the African population and the European population for allele 1 falling into the allele frequency bin between 0.15-0.20 in the African population; (c) comparisons between the African population and the South Asian population for allele 1 falling into the allele frequency bin between 0.15-0.20 in the African population; (d) comparisons between the East Asian population and the African population for allele 1 falling into the allele frequency bin between 0.15-0.20 in the East Asian population; (e) comparisons between the East Asian population and the European population for allele 1 falling into the allele frequency bin between 0.15-0.20 in the East Asian population; (f) comparisons between the East Asian population and the South Asian population for allele 1 falling into the allele frequency bin between 0.15-0.20 in the East Asian population; (g) comparisons between the European population and the African population for allele 1 falling into the allele frequency bin between 0.15-0.20 in the European population; (h) comparisons between the European population and the East Asian population for allele 1 falling into the allele frequency bin between 0.15-0.20 in the European population; (i) comparisons between the European population and the South Asian population for allele 1 falling into the allele frequency bin between 0.15-0.20 in the European population; (j) comparisons between the South Asian population and the African population for allele 1 falling into the allele frequency bin between 0.15-0.20 in the South Asian population; (k) comparisons between the South Asian population and the East Asian population for allele 1 falling into the allele frequency bin between 0.15-0.20 in the South Asian population; (l) comparisons between the South Asian population and the European population for allele 1 falling into the allele frequency bin between 0.15-0.20 in the South Asian population.

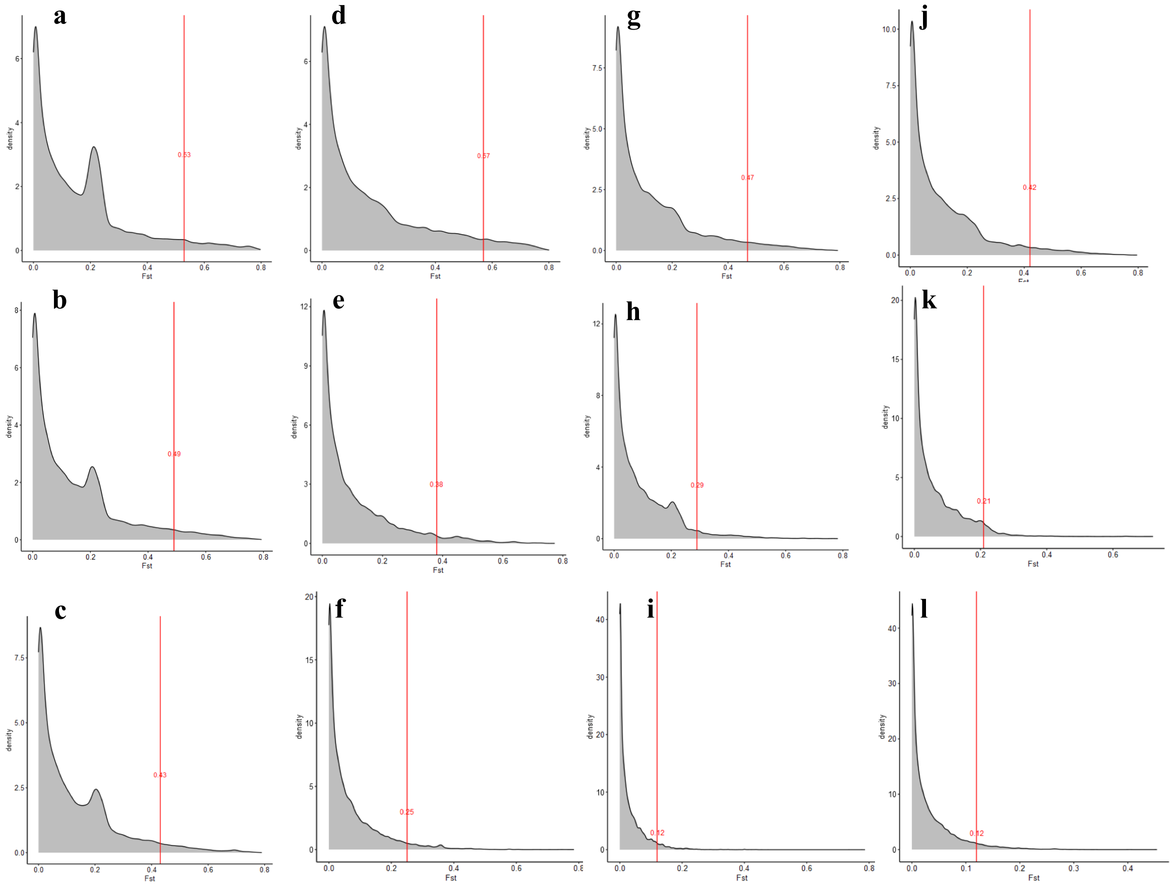

Figure S4 Pairwise F_{ST} distributions. The top 5% thresholds were given. (a) comparisons between the African population and the East Asian population for allele 1 falling into the allele frequency bin between 0.20-0.25 in the African population; (b) comparisons between the African population and the European population for allele 1 falling into the allele frequency bin between 0.20-0.25 in the African population; (c) comparisons between the African population and the South Asian population for allele 1 falling into the allele frequency bin between 0.20-0.25 in the African population; (d) comparisons between the East Asian population and the African population for allele 1 falling into the allele frequency bin between 0.20-0.25 in the East Asian population; (e) comparisons between the East Asian population and the European population for allele 1 falling into the allele frequency bin between 0.20-0.25 in the East Asian population; (f) comparisons between the East Asian population and the South Asian population for allele 1 falling into the allele frequency bin between 0.20-0.25 in the East Asian population; (g) comparisons between the European population and the African population for allele 1 falling into the allele frequency bin between 0.20-0.25 in the European population; (h) comparisons between the European population and the East Asian population for allele 1 falling into the allele frequency bin between 0.20-0.25 in the European population; (i) comparisons between the European population and the South Asian population for allele 1 falling into the allele frequency bin between 0.20-0.25 in the European population; (j) comparisons between the South Asian population and the African population for allele 1 falling into the allele frequency bin between 0.20-0.25 in the South Asian population; (k) comparisons between the South Asian population and the East Asian population for allele 1 falling into the allele frequency bin between 0.20-0.25 in the South Asian population; (l) comparisons between the South Asian population and the European population for allele 1 falling into the allele frequency bin between 0.20-0.25 in the South Asian population.

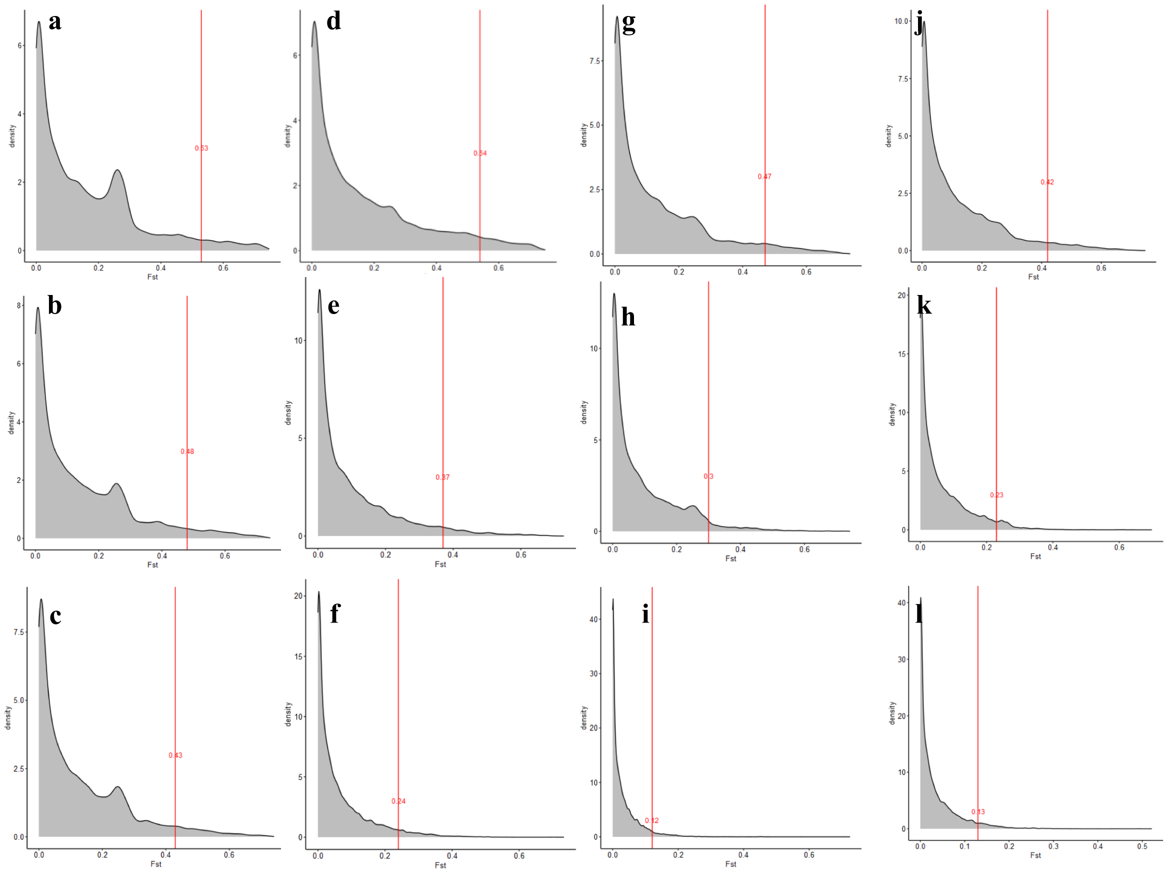

Figure S5 Pairwise F_{ST} distributions. The top 5% thresholds were given. (a) comparisons between the African population and the East Asian population for allele 1 falling into the allele frequency bin between 0.25-0.30 in the African population; (b) comparisons between the African population and the European population for allele 1 falling into the allele frequency bin between 0.25-0.30 in the African population; (c) comparisons between the African population and the South Asian population for allele 1 falling into the allele frequency bin between 0.25-0.30 in the African population; (d) comparisons between the East Asian population and the African population for allele 1 falling into the allele frequency bin between 0.25-0.30 in the East Asian population; (e) comparisons between the East Asian population and the European population for allele 1 falling into the allele frequency bin between 0.25-0.30 in the East Asian population; (f) comparisons between the East Asian population and the South Asian population for allele 1 falling into the allele frequency bin between 0.25-0.30 in the East Asian population; (g) comparisons between the European population and the African population for allele 1 falling into the allele frequency bin between 0.25-0.30 in the European population; (h) comparisons between the European population and the East Asian population for allele 1 falling into the allele frequency bin between 0.25-0.30 in the European population; (i) comparisons between the European population and the South Asian population for allele 1 falling into the allele frequency bin between 0.25-0.30 in the European population; (j) comparisons between the South Asian population and the African population for allele 1 falling into the allele frequency bin between 0.25-0.30 in the South Asian population; (k) comparisons between the South Asian population and the East Asian population for allele 1 falling into the allele frequency bin between 0.25-0.30 in the South Asian population; (l) comparisons between the South Asian population and the European population for allele 1 falling into the allele frequency bin between 0.25-0.30 in the South Asian population.

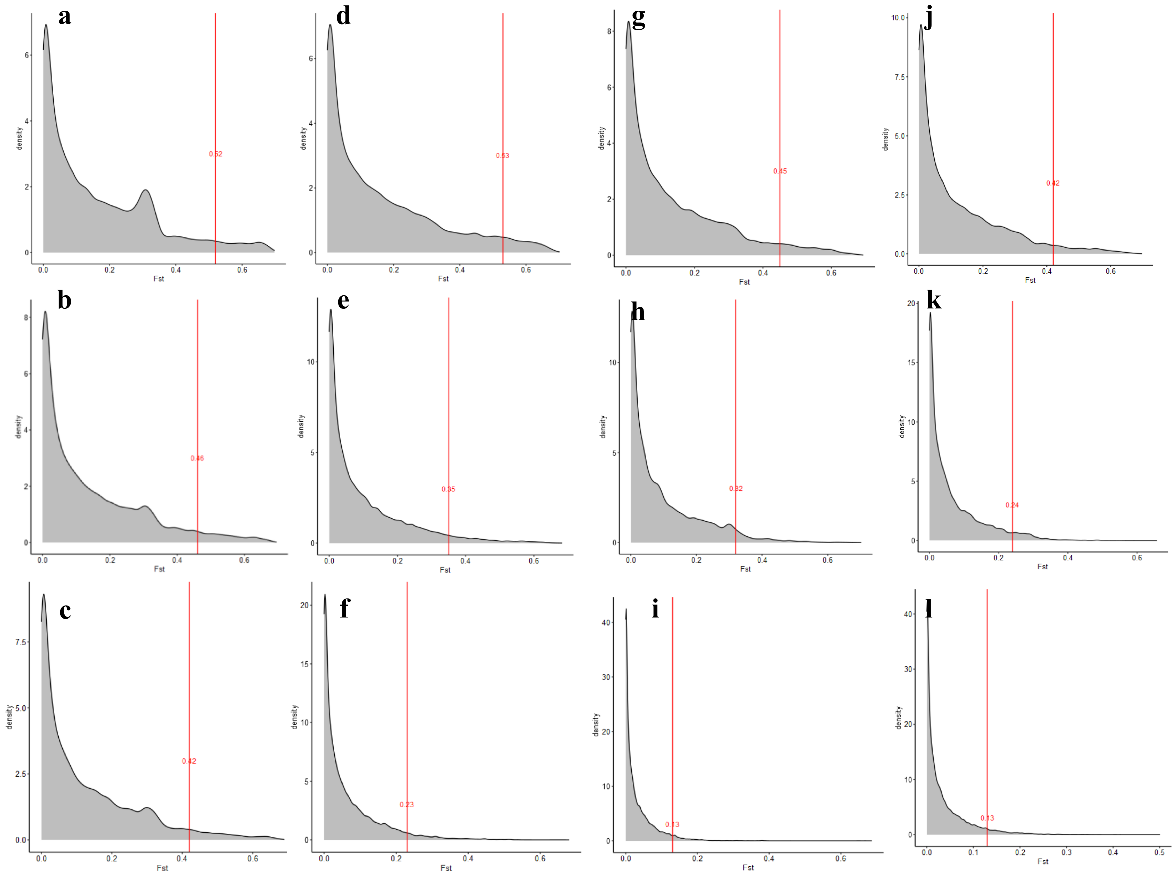

Figure S6 Pairwise F_{ST} distributions. The top 5% thresholds were given. (a) comparisons between the African population and the East Asian population for allele 1 falling into the allele frequency bin between 0.30-0.35 in the African population; (b) comparisons between the African population and the European population for allele 1 falling into the allele frequency bin between 0.30-0.35 in the African population; (c) comparisons between the African population and the South Asian population for allele 1 falling into the allele frequency bin between 0.30-0.35 in the African population; (d) comparisons between the East Asian population and the African population for allele 1 falling into the allele frequency bin between 0.30-0.35 in the East Asian population; (e) comparisons between the East Asian population and the European population for allele 1 falling into the allele frequency bin between 0.30-0.35 in the East Asian population; (f) comparisons between the East Asian population and the South Asian population for allele 1 falling into the allele frequency bin between 0.30-0.35 in the East Asian population; (g) comparisons between the European population and the African population for allele 1 falling into the allele frequency bin between 0.30-0.35 in the European population; (h) comparisons between the European population and the East Asian population for allele 1 falling into the allele frequency bin between 0.30-0.35 in the European population; (i) comparisons between the European population and the South Asian population for allele 1 falling into the allele frequency bin between 0.30-0.35 in the European population; (j) comparisons between the South Asian population and the African population for allele 1 falling into the allele frequency bin between 0.30-0.35 in the South Asian population; (k) comparisons between the South Asian population and the East Asian population for allele 1 falling into the allele frequency bin between 0.30-0.35 in the South Asian population; (l) comparisons between the South Asian population and the European population for allele 1 falling into the allele frequency bin between 0.30-0.35 in the South Asian population.

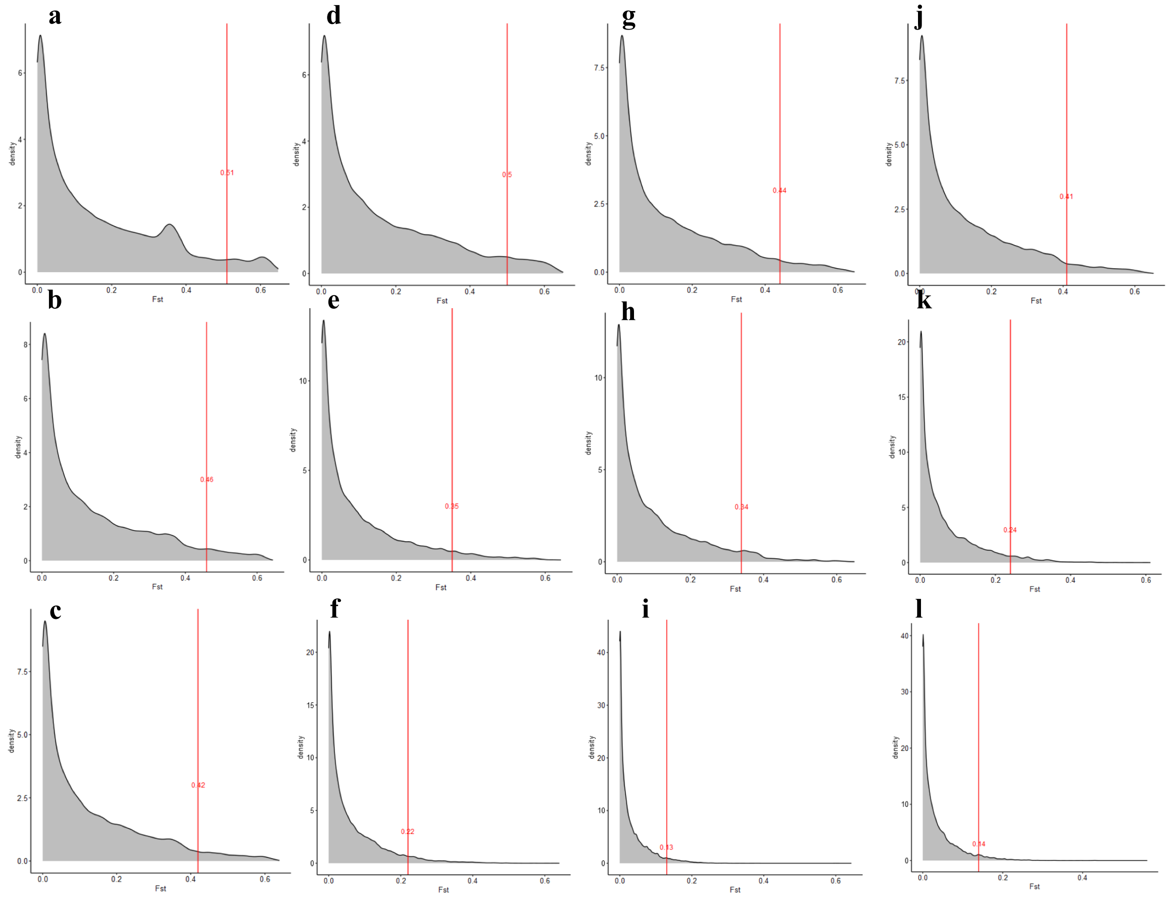

Figure S7 Pairwise F_{ST} distributions. The top 5% thresholds were given. (a) comparisons between the African population and the East Asian population for allele 1 falling into the allele frequency bin between 0.35-0.40 in the African population; (b) comparisons between the African population and the European population for allele 1 falling into the allele frequency bin between 0.35-0.40 in the African population; (c) comparisons between the African population and the South Asian population for allele 1 falling into the allele frequency bin between 0.35-0.40 in the African population; (d) comparisons between the East Asian population and the African population for allele 1 falling into the allele frequency bin between 0.35-0.40 in the East Asian population; (e) comparisons between the East Asian population and the European population for allele 1 falling into the allele frequency bin between 0.35-0.40 in the East Asian population; (f) comparisons between the East Asian population and the South Asian population for allele 1 falling into the allele frequency bin between 0.35-0.40 in the East Asian population; (g) comparisons between the European population and the African population for allele 1 falling into the allele frequency bin between 0.35-0.40 in the European population; (h) comparisons between the European population and the East Asian population for allele 1 falling into the allele frequency bin between 0.35-0.40 in the European population; (i) comparisons between the European population and the South Asian population for allele 1 falling into the allele frequency bin between 0.35-0.40 in the European population; (j) comparisons between the South Asian population and the African population for allele 1 falling into the allele frequency bin between 0.35-0.40 in the South Asian population; (k) comparisons between the South Asian population and the East Asian population for allele 1 falling into the allele frequency bin between 0.35-0.40 in the South Asian population; (l) comparisons between the South Asian population and the European population for allele 1 falling into the allele frequency bin between 0.35-0.40 in the South Asian population.

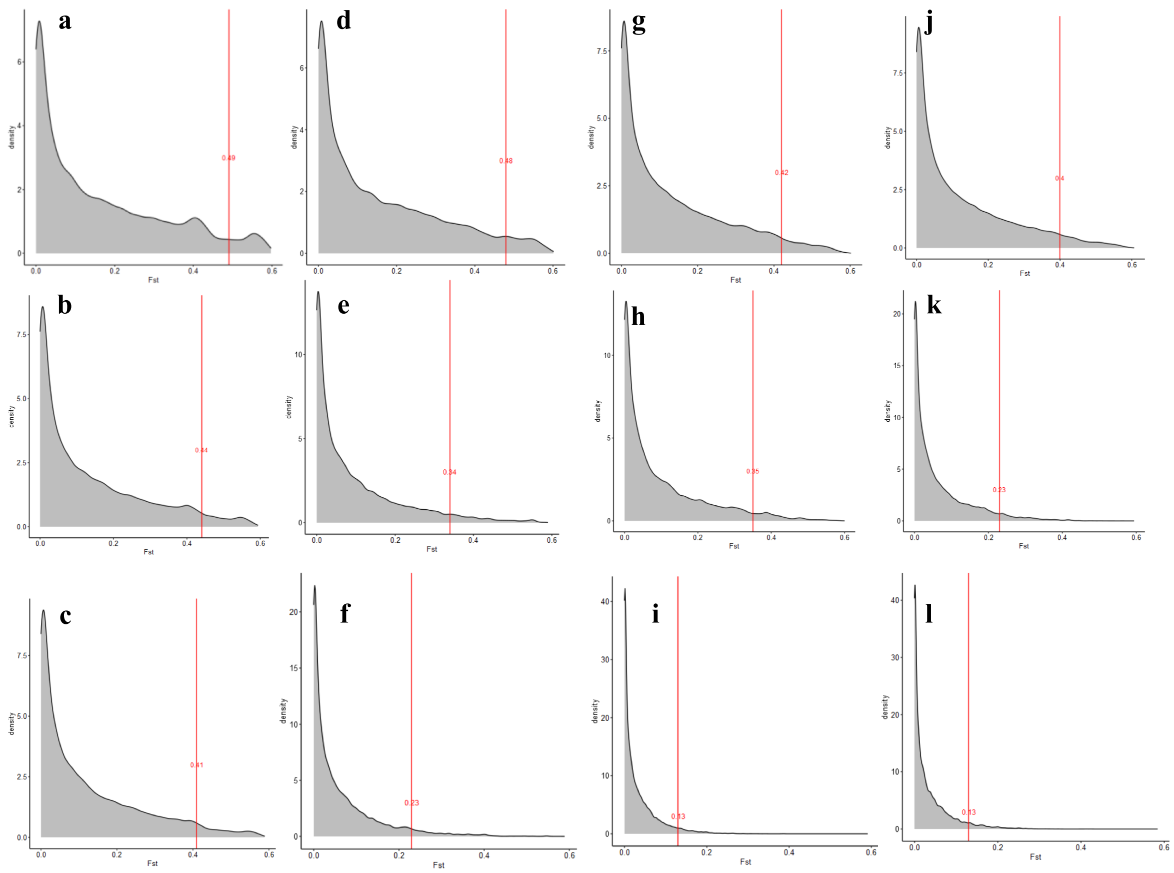

Figure S8 Pairwise F_{ST} distributions. The top 5% thresholds were given. (a) comparisons between the African population and the East Asian population for allele 1 falling into the allele frequency bin between 0.40-0.45 in the African population; (b) comparisons between the African population and the European population for allele 1 falling into the allele frequency bin between 0.40-0.45 in the African population; (c) comparisons between the African population and the South Asian population for allele 1 falling into the allele frequency bin between 0.40-0.45 in the African population; (d) comparisons between the East Asian population and the African population for allele 1 falling into the allele frequency bin between 0.40-0.45 in the East Asian population; (e) comparisons between the East Asian population and the European population for allele 1 falling into the allele frequency bin between 0.40-0.45 in the East Asian population; (f) comparisons between the East Asian population and the South Asian population for allele 1 falling into the allele frequency bin between 0.40-0.45 in the East Asian population; (g) comparisons between the European population and the African population for allele 1 falling into the allele frequency bin between 0.40-0.45 in the European population; (h) comparisons between the European population and the East Asian population for allele 1 falling into the allele frequency bin between 0.40-0.45 in the European population; (i) comparisons between the European population and the South Asian population for allele 1 falling into the allele frequency bin between 0.40-0.45 in the European population; (j) comparisons between the South Asian population and the African population for allele 1 falling into the allele frequency bin between 0.40-0.45 in the South Asian population; (k) comparisons between the South Asian population and the East Asian population for allele 1 falling into the allele frequency bin between 0.40-0.45 in the South Asian population; (l) comparisons between the South Asian population and the European population for allele 1 falling into the allele frequency bin between 0.40-0.45 in the South Asian population.

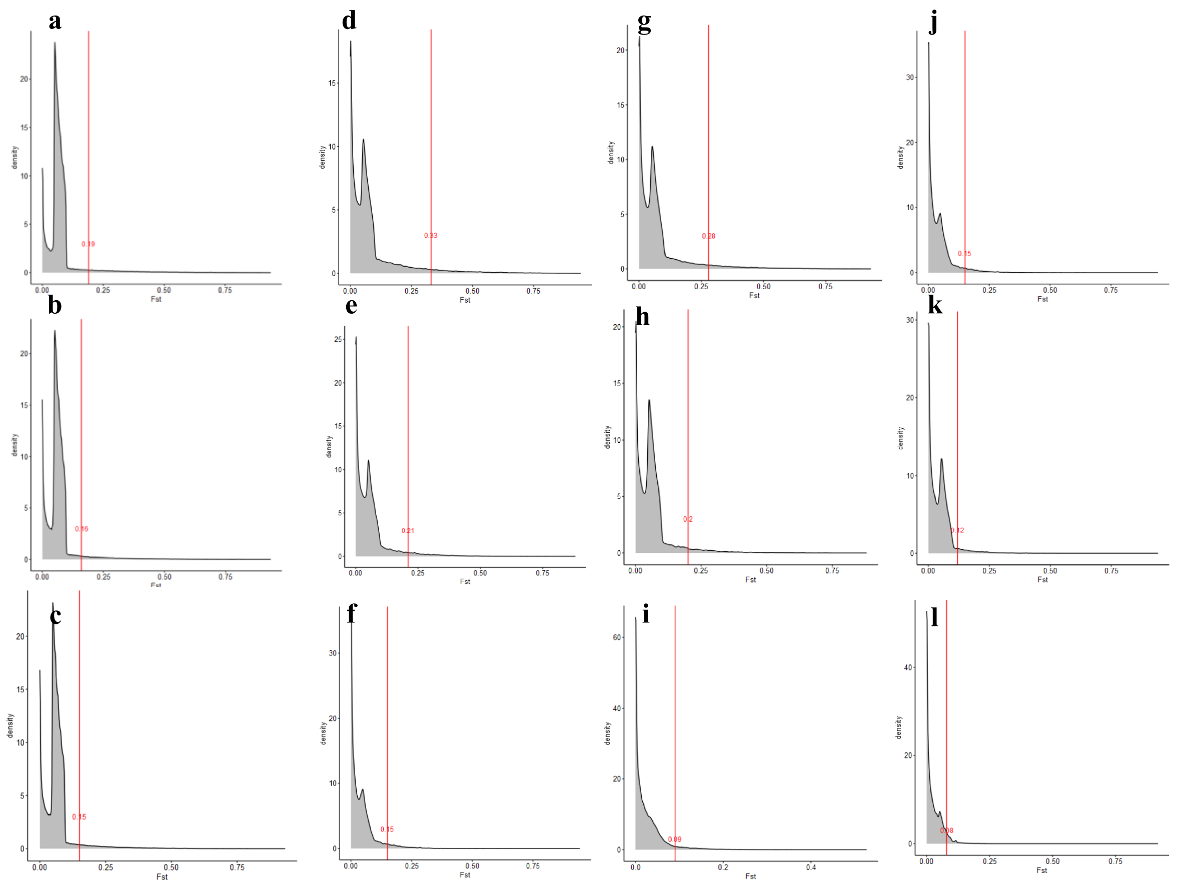

Figure S9 Pairwise F_{ST} distributions. The top 5% thresholds were given. (a) comparisons between the African population and the East Asian population for allele 2 falling into the allele frequency bin between 0.05-0.10 in the African population; (b) comparisons between the African population and the European population for allele 2 falling into the allele frequency bin between 0.05-0.10 in the African population; (c) comparisons between the African population and the South Asian population for allele 2 falling into the allele frequency bin between 0.05-0.10 in the African population; (d) comparisons between the East Asian population and the African population for allele 2 falling into the allele frequency bin between 0.05-0.10 in the East Asian population; (e) comparisons between the East Asian population and the European population for allele 2 falling into the allele frequency bin between 0.05-0.10 in the East Asian population; (f) comparisons between the East Asian population and the South Asian population for allele 2 falling into the allele frequency bin between 0.05-0.10 in the East Asian population; (g) comparisons between the European population and the African population for allele 2 falling into the allele frequency bin between 0.05-0.10 in the European population; (h) comparisons between the European population and the East Asian population for allele 2 falling into the allele frequency bin between 0.05-0.10 in the European population; (i) comparisons between the European population and the South Asian population for allele 2 falling into the allele frequency bin between 0.05-0.10 in the European population; (j) comparisons between the South Asian population and the African population for allele 2 falling into the allele frequency bin between 0.05-0.10 in the South Asian population; (k) comparisons between the South Asian population and the East Asian population for allele 2 falling into the allele frequency bin between 0.05-0.10 in the South Asian population; (l) comparisons between the South Asian population and the European population for allele 2 falling into the allele frequency bin between 0.05-0.10 in the South Asian population.

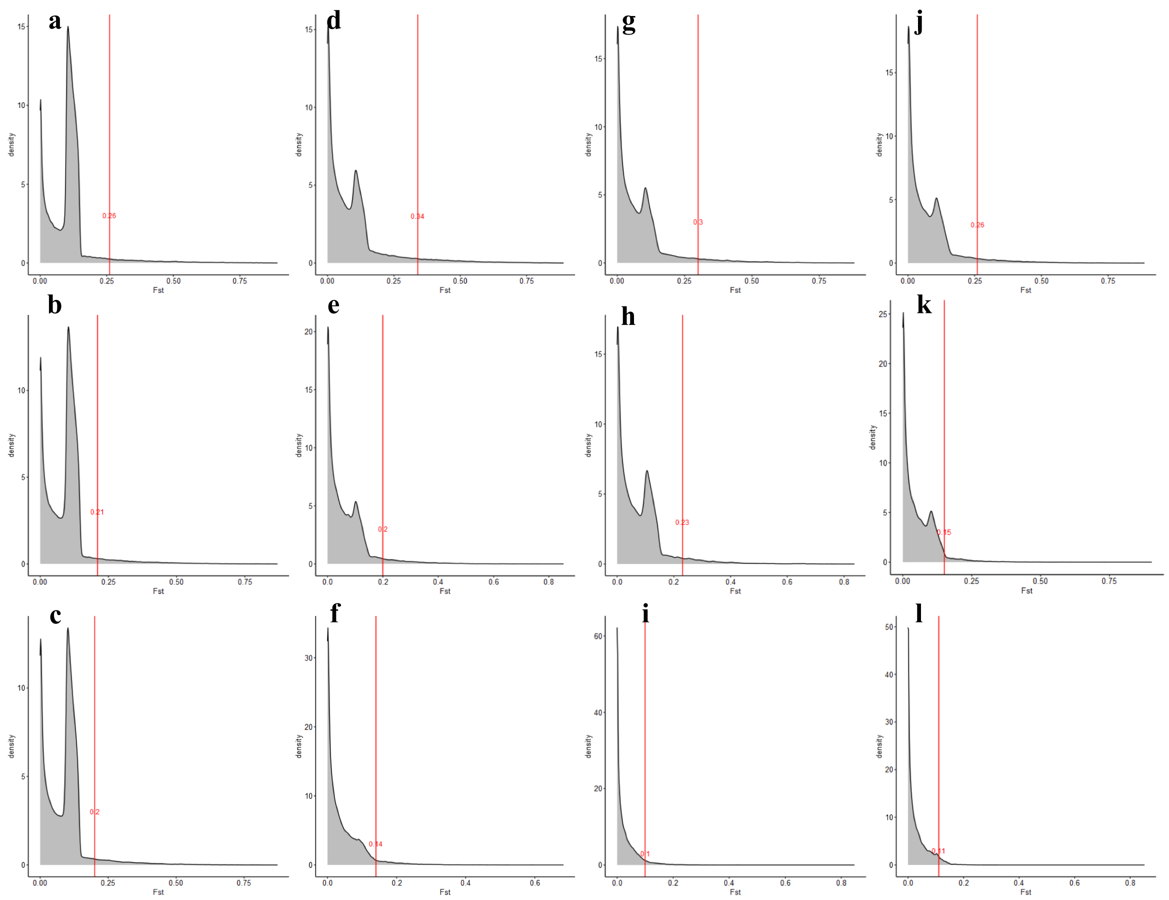

Figure S10 Pairwise F_{ST} distributions. The top 5% thresholds were given. (a) comparisons between the African population and the East Asian population for allele 2 falling into the allele frequency bin between 0.10-0.15 in the African population; (b) comparisons between the African population and the European population for allele 2 falling into the allele frequency bin between 0.10-0.15 in the African population; (c) comparisons between the African population and the South Asian population for allele 2 falling into the allele frequency bin between 0.10-0.15 in the African population; (d) comparisons between the East Asian population and the African population for allele 2 falling into the allele frequency bin between 0.10-0.15 in the East Asian population; (e) comparisons between the East Asian population and the European population for allele 2 falling into the allele frequency bin between 0.10-0.15 in the East Asian population; (f) comparisons between the East Asian population and the South Asian population for allele 2 falling into the allele frequency bin between 0.10-0.15 in the East Asian population; (g) comparisons between the European population and the African population for allele 2 falling into the allele frequency bin between 0.10-0.15 in the European population; (h) comparisons between the European population and the East Asian population for allele 2 falling into the allele frequency bin between 0.10-0.15 in the European population; (i) comparisons between the European population and the South Asian population for allele 2 falling into the allele frequency bin between 0.10-0.15 in the European population; (j) comparisons between the South Asian population and the African population for allele 2 falling into the allele frequency bin between 0.10-0.15 in the South Asian population; (k) comparisons between the South Asian population and the East Asian population for allele 2 falling into the allele frequency bin between 0.10-0.15 in the South Asian population; (l) comparisons between the South Asian population and the European population for allele 2 falling into the allele frequency bin between 0.10-0.15 in the South Asian population.

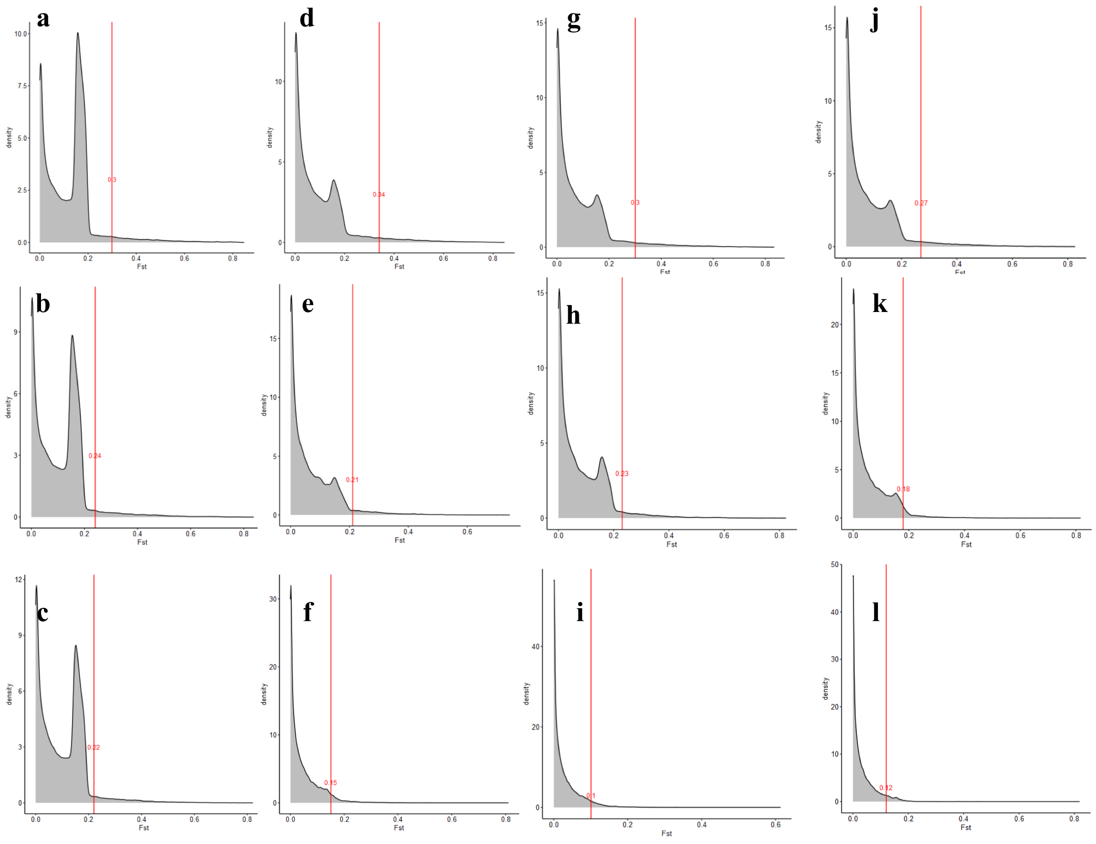

Figure S11 Pairwise F_{ST} distributions. The top 5% thresholds were given. (a) comparisons between the African population and the East Asian population for allele 2 falling into the allele frequency bin between 0.15-0.20 in the African population; (b) comparisons between the African population and the European population for allele 2 falling into the allele frequency bin between 0.15-0.20 in the African population; (c) comparisons between the African population and the South Asian population for allele 2 falling into the allele frequency bin between 0.15-0.20 in the African population; (d) comparisons between the East Asian population and the African population for allele 2 falling into the allele frequency bin between 0.15-0.20 in the East Asian population; (e) comparisons between the East Asian population and the European population for allele 2 falling into the allele frequency bin between 0.15-0.20 in the East Asian population; (f) comparisons between the East Asian population and the South Asian population for allele 2 falling into the allele frequency bin between 0.15-0.20 in the East Asian population; (g) comparisons between the European population and the African population for allele 2 falling into the allele frequency bin between 0.15-0.20 in the European population; (h) comparisons between the European population and the East Asian population for allele 2 falling into the allele frequency bin between 0.15-0.20 in the European population; (i) comparisons between the European population and the South Asian population for allele 2 falling into the allele frequency bin between 0.15-0.20 in the European population; (j) comparisons between the South Asian population and the African population for allele 2 falling into the allele frequency bin between 0.15-0.20 in the South Asian population; (k) comparisons between the South Asian population and the East Asian population for allele 2 falling into the allele frequency bin between 0.15-0.20 in the South Asian population; (l) comparisons between the South Asian population and the European population for allele 2 falling into the allele frequency bin between 0.15-0.20 in the South Asian population.

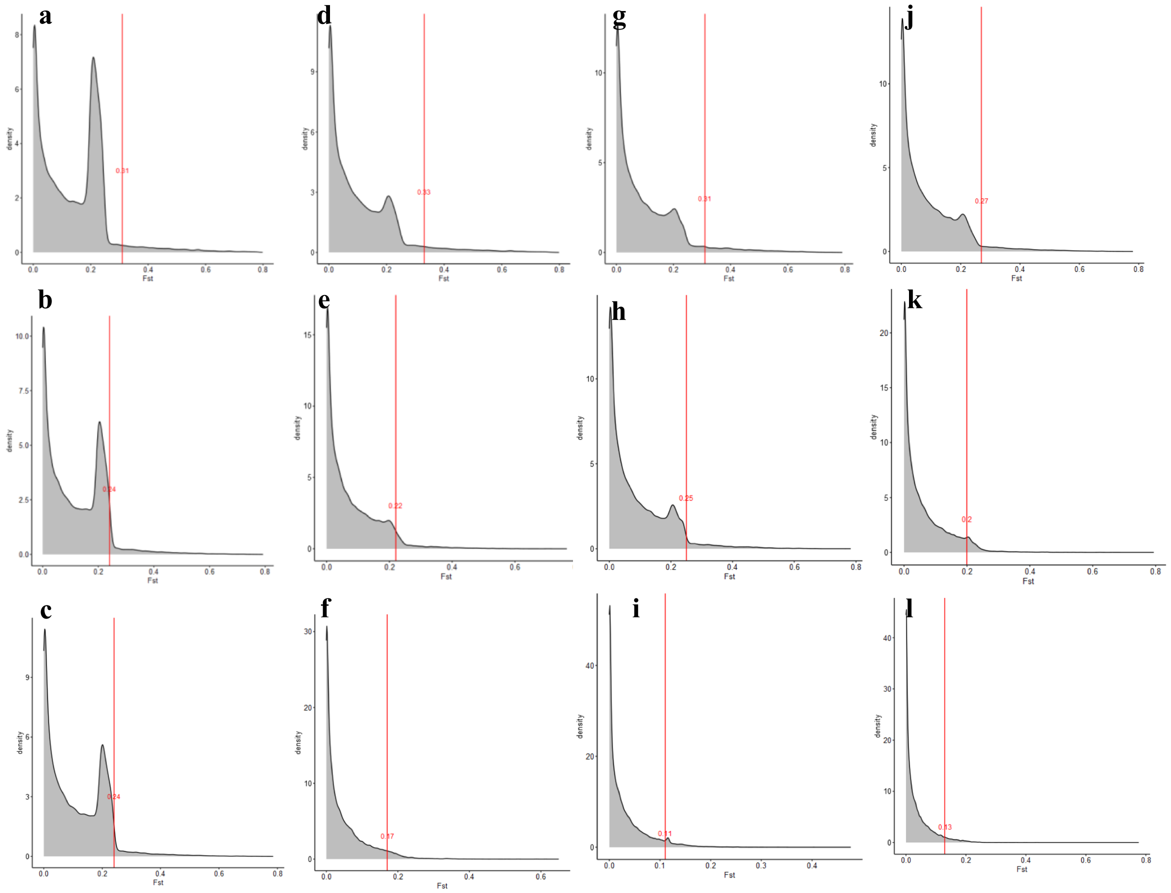

Figure S12 Pairwise F_{ST} distributions. The top 5% thresholds were given. (a) comparisons between the African population and the East Asian population for allele 2 falling into the allele frequency bin between 0.20-0.25 in the African population; (b) comparisons between the African population and the European population for allele 2 falling into the allele frequency bin between 0.20-0.25 in the African population; (c) comparisons between the African population and the South Asian population for allele 2 falling into the allele frequency bin between 0.20-0.25 in the African population; (d) comparisons between the East Asian population and the African population for allele 2 falling into the allele frequency bin between 0.20-0.25 in the East Asian population; (e) comparisons between the East Asian population and the European population for allele 2 falling into the allele frequency bin between 0.20-0.25 in the East Asian population; (f) comparisons between the East Asian population and the South Asian population for allele 2 falling into the allele frequency bin between 0.20-0.25 in the East Asian population; (g) comparisons between the European population and the African population for allele 2 falling into the allele frequency bin between 0.20-0.25 in the European population; (h) comparisons between the European population and the East Asian population for allele 2 falling into the allele frequency bin between 0.20-0.25 in the European population; (i) comparisons between the European population and the South Asian population for allele 2 falling into the allele frequency bin between 0.20-0.25 in the European population; (j) comparisons between the South Asian population and the African population for allele 2 falling into the allele frequency bin between 0.20-0.25 in the South Asian population; (k) comparisons between the South Asian population and the East Asian population for allele 2 falling into the allele frequency bin between 0.20-0.25 in the South Asian population; (l) comparisons between the South Asian population and the European population for allele 2 falling into the allele frequency bin between 0.20-0.25 in the South Asian population.

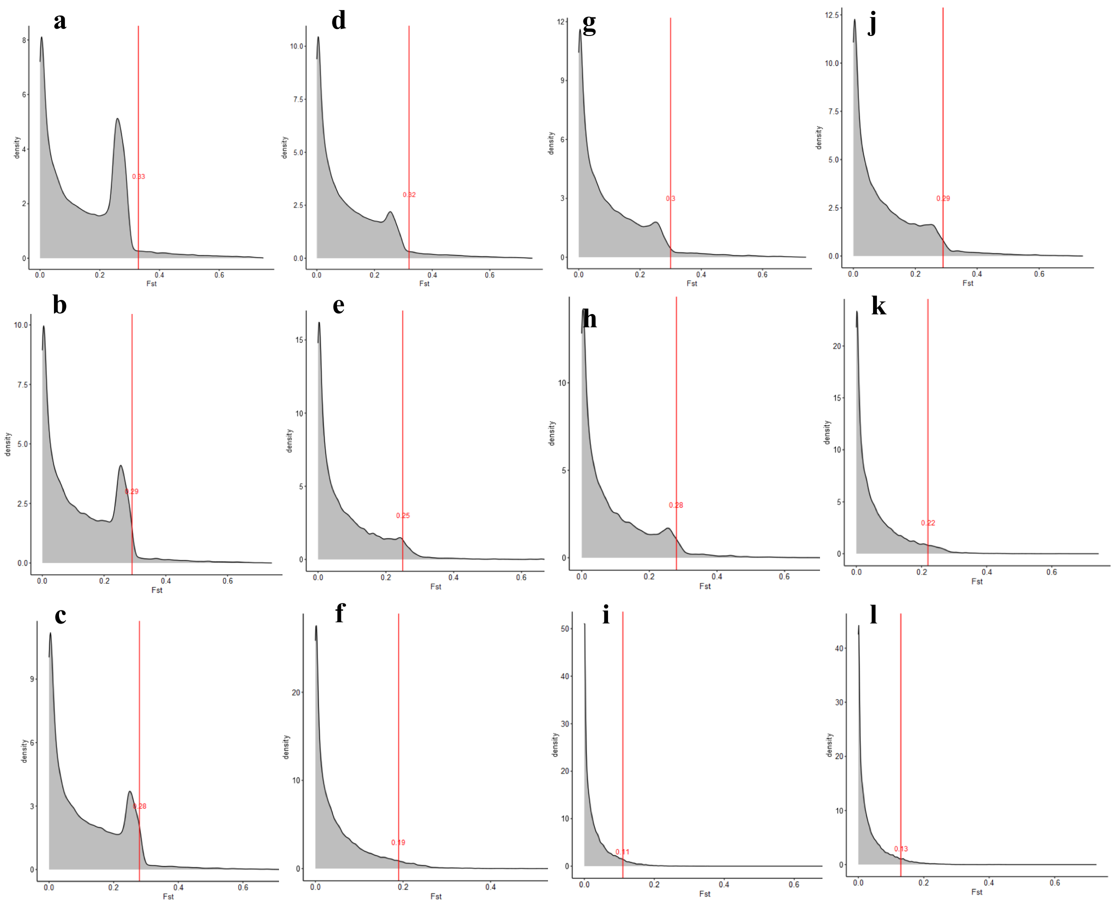

Figure S13 Pairwise F_{ST} distributions. The top 5% thresholds were given. (a) comparisons between the African population and the East Asian population for allele 2 falling into the allele frequency bin between 0.25-0.30 in the African population; (b) comparisons between the African population and the European population for allele 2 falling into the allele frequency bin between 0.25-0.30 in the African population; (c) comparisons between the African population and the South Asian population for allele 2 falling into the allele frequency bin between 0.25-0.30 in the African population; (d) comparisons between the East Asian population and the African population for allele 2 falling into the allele frequency bin between 0.25-0.30 in the East Asian population; (e) comparisons between the East Asian population and the European population for allele 2 falling into the allele frequency bin between 0.25-0.30 in the East Asian population; (f) comparisons between the East Asian population and the South Asian population for allele 2 falling into the allele frequency bin between 0.25-0.30 in the East Asian population; (g) comparisons between the European population and the African population for allele 2 falling into the allele frequency bin between 0.25-0.30 in the European population; (h) comparisons between the European population and the East Asian population for allele 2 falling into the allele frequency bin between 0.25-0.30 in the European population; (i) comparisons between the European population and the South Asian population for allele 2 falling into the allele frequency bin between 0.25-0.30 in the European population; (j) comparisons between the South Asian population and the African population for allele 2 falling into the allele frequency bin between 0.25-0.30 in the South Asian population; (k) comparisons between the South Asian population and the East Asian population for allele 2 falling into the allele frequency bin between 0.25-0.30 in the South Asian population; (l) comparisons between the South Asian population and the European population for allele 2 falling into the allele frequency bin between 0.25-0.30 in the South Asian population.

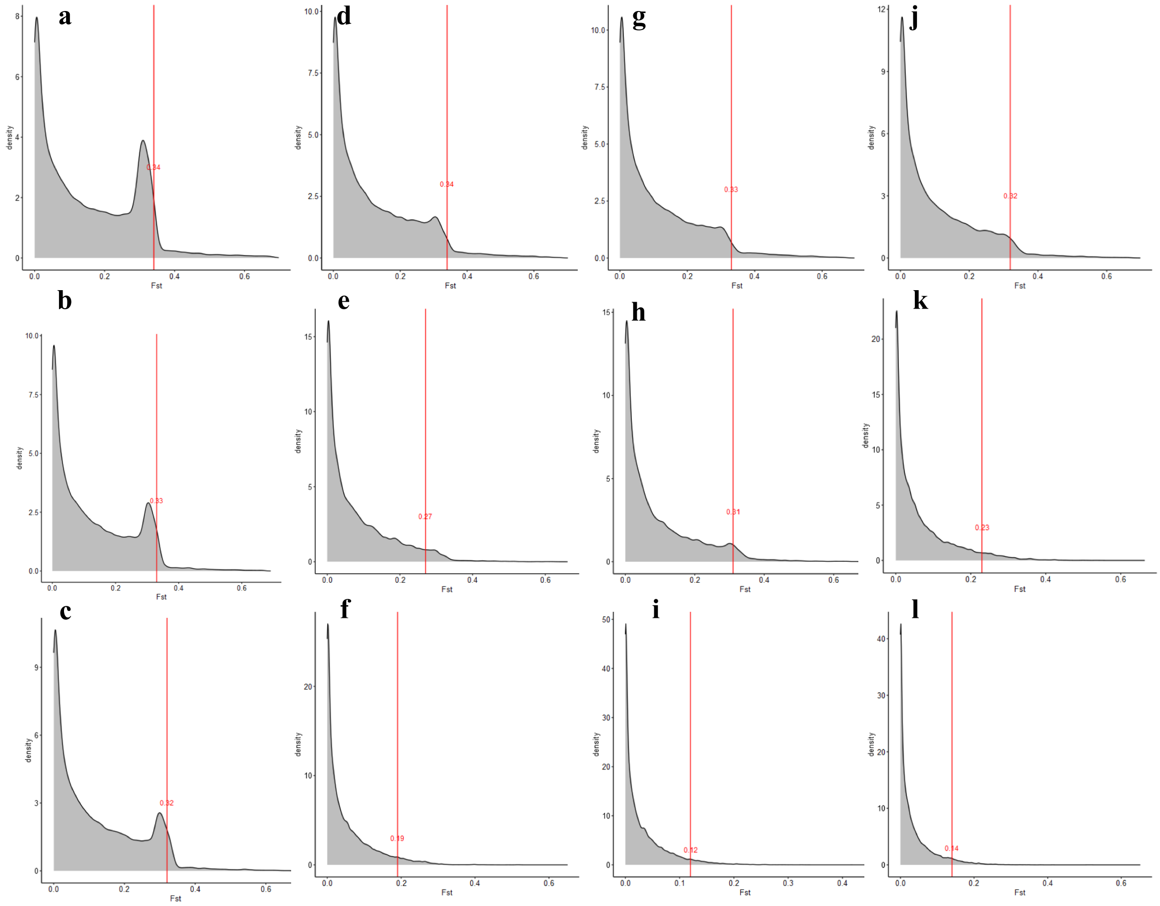

Figure S14 Pairwise F_{ST} distributions. The top 5% thresholds were given. (a) comparisons between the African population and the East Asian population for allele 2 falling into the allele frequency bin between 0.30-0.35 in the African population; (b) comparisons between the African population and the European population for allele 2 falling into the allele frequency bin between 0.30-0.35 in the African population; (c) comparisons between the African population and the South Asian population for allele 2 falling into the allele frequency bin between 0.30-0.35 in the African population; (d) comparisons between the East Asian population and the African population for allele 2 falling into the allele frequency bin between 0.30-0.35 in the East Asian population; (e) comparisons between the East Asian population and the European population for allele 2 falling into the allele frequency bin between 0.30-0.35 in the East Asian population; (f) comparisons between the East Asian population and the South Asian population for allele 2 falling into the allele frequency bin between 0.30-0.35 in the East Asian population; (g) comparisons between the European population and the African population for allele 2 falling into the allele frequency bin between 0.30-0.35 in the European population; (h) comparisons between the European population and the East Asian population for allele 2 falling into the allele frequency bin between 0.30-0.35 in the European population; (i) comparisons between the European population and the South Asian population for allele 2 falling into the allele frequency bin between 0.30-0.35 in the European population; (j) comparisons between the South Asian population and the African population for allele 2 falling into the allele frequency bin between 0.30-0.35 in the South Asian population; (k) comparisons between the South Asian population and the East Asian population for allele 2 falling into the allele frequency bin between 0.30-0.35 in the South Asian population; (l) comparisons between the South Asian population and the European population for allele 2 falling into the allele frequency bin between 0.30-0.35 in the South Asian population.

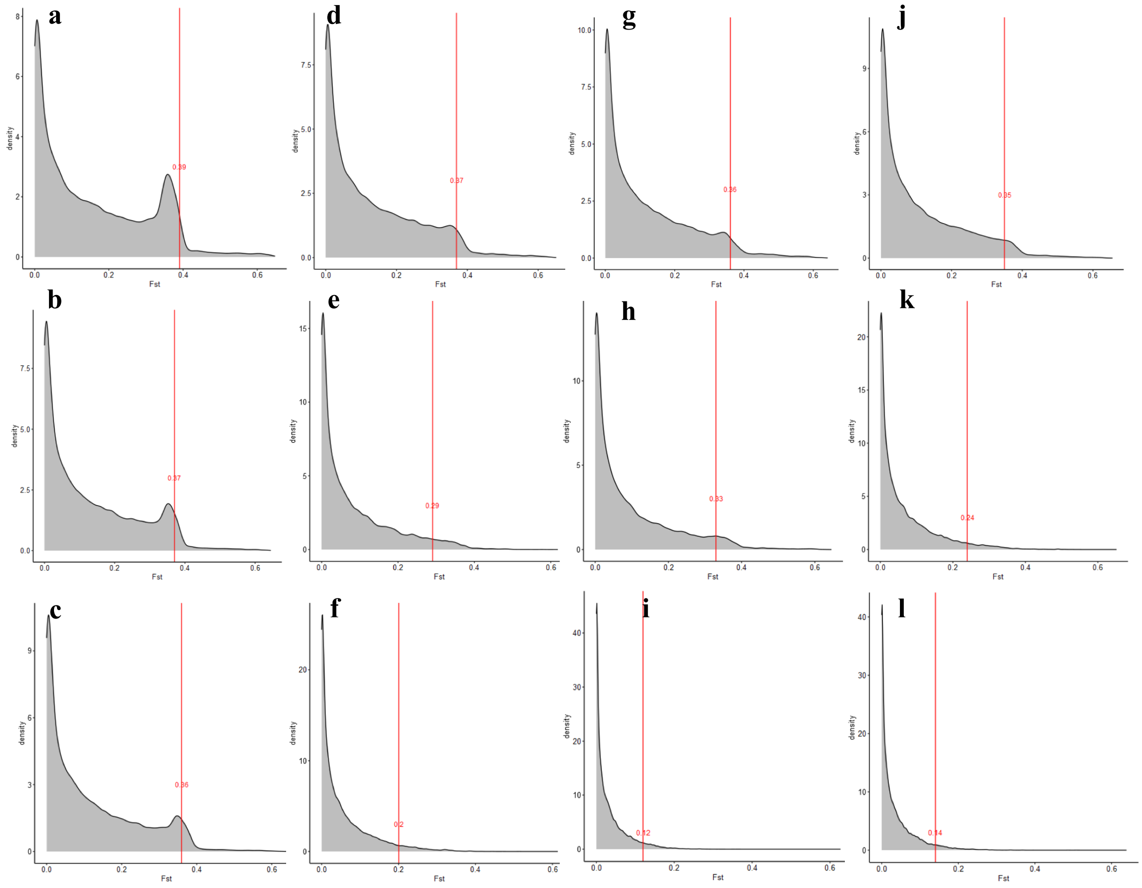

Figure S15 Pairwise F_{ST} distributions. The top 5% thresholds were given. (a) comparisons between the African population and the East Asian population for allele 2 falling into the allele frequency bin between 0.35-0.40 in the African population; (b) comparisons between the African population and the European population for allele 2 falling into the allele frequency bin between 0.35-0.40 in the African population; (c) comparisons between the African population and the South Asian population for allele 2 falling into the allele frequency bin between 0.35-0.40 in the African population; (d) comparisons between the East Asian population and the African population for allele 2 falling into the allele frequency bin between 0.35-0.40 in the East Asian population; (e) comparisons between the East Asian population and the European population for allele 2 falling into the allele frequency bin between 0.35-0.40 in the East Asian population; (f) comparisons between the East Asian population and the South Asian population for allele 2 falling into the allele frequency bin between 0.35-0.40 in the East Asian population; (g) comparisons between the European population and the African population for allele 2 falling into the allele frequency bin between 0.35-0.40 in the European population; (h) comparisons between the European population and the East Asian population for allele 2 falling into the allele frequency bin between 0.35-0.40 in the European population; (i) comparisons between the European population and the South Asian population for allele 2 falling into the allele frequency bin between 0.35-0.40 in the European population; (j) comparisons between the South Asian population and the African population for allele 2 falling into the allele frequency bin between 0.35-0.40 in the South Asian population; (k) comparisons between the South Asian population and the East Asian population for allele 2 falling into the allele frequency bin between 0.35-0.40 in the South Asian population; (l) comparisons between the South Asian population and the European population for allele 2 falling into the allele frequency bin between 0.35-0.40 in the South Asian population.

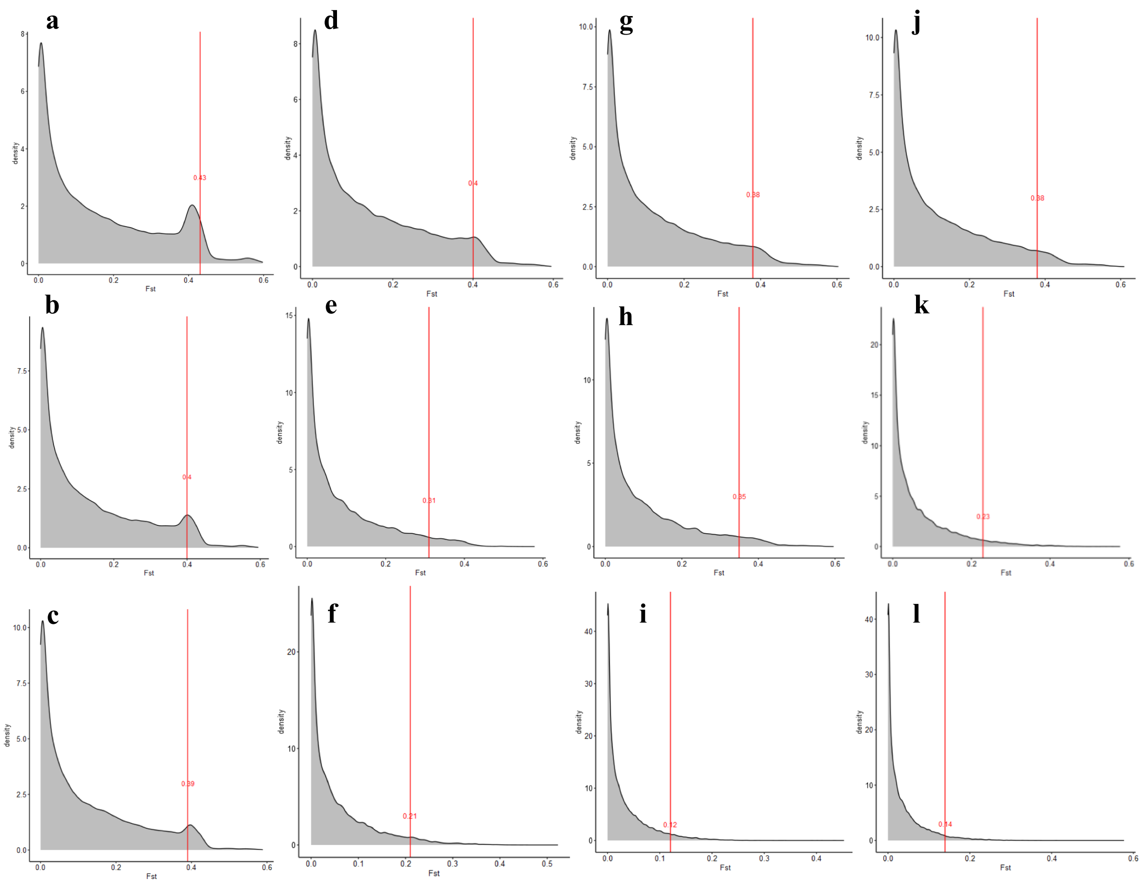

Figure S16 Pairwise F_{ST} distributions. The top 5% thresholds were given. (a) comparisons between the African population and the East Asian population for allele 2 falling into the allele frequency bin between 0.40-0.45 in the African population; (b) comparisons between the African population and the European population for allele 2 falling into the allele frequency bin between 0.40-0.45 in the African population; (c) comparisons between the African population and the South Asian population for allele 2 falling into the allele frequency bin between 0.40-0.45 in the African population; (d) comparisons between the East Asian population and the African population for allele 2 falling into the allele frequency bin between 0.40-0.45 in the East Asian population; (e) comparisons between the East Asian population and the European population for allele 2 falling into the allele frequency bin between 0.40-0.45 in the East Asian population; (f) comparisons between the East Asian population and the South Asian population for allele 2 falling into the allele frequency bin between 0.40-0.45 in the East Asian population; (g) comparisons between the European population and the African population for allele 2 falling into the allele frequency bin between 0.40-0.45 in the European population; (h) comparisons between the European population and the East Asian population for allele 2 falling into the allele frequency bin between 0.40-0.45 in the European population; (i) comparisons between the European population and the South Asian population for allele 2 falling into the allele frequency bin between 0.40-0.45 in the European population; (j) comparisons between the South Asian population and the African population for allele 2 falling into the allele frequency bin between 0.40-0.45 in the South Asian population; (k) comparisons between the South Asian population and the East Asian population for allele 2 falling into the allele frequency bin between 0.40-0.45 in the South Asian population; (l) comparisons between the South Asian population and the European population for allele 2 falling into the allele frequency bin between 0.40-0.45 in the South Asian population.
