# Supplementary material for: The adaptive evolution of cancer driver genes
Source: BMC Genomics. 2023 Apr 25;24:215. doi: 10.1186/s12864-023-09301-9 (PMC10131384; doi:10.1186/s12864-023-09301-9)
Supplement: Supplementary file 3 — Additional file 3. [file 12864_2023_9301_MOESM3_ESM.pdf]

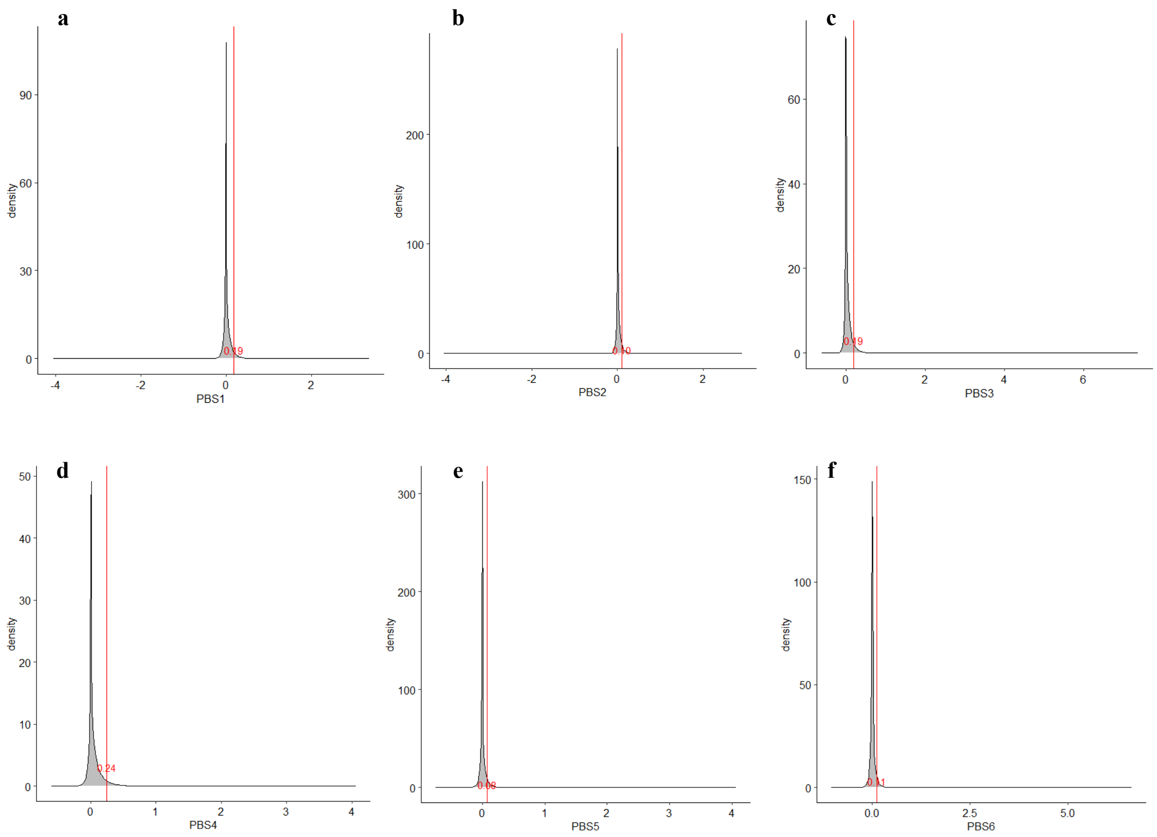

Figure S1 PBS distributions of various comparisons between populations, with the African population as the outgroup. (a) PBS1, divergence of the European population from the East Asian population; (b) PBS2, divergence of the European population from the South Asian population; (c) PBS3, divergence of the East Asian population from the South Asian population; (d) PBS4, divergence of the East Asian population from the European population; (e) PBS5, divergence of the South Asian population from the European population; (f) PBS6, divergence of the South Asian population from the East Asian population. Top 5% values were set as the thresholds.
