# Supplementary material for: The adaptive evolution of cancer driver genes
Source: BMC Genomics. 2023 Apr 25;24:215. doi: 10.1186/s12864-023-09301-9 (PMC10131384; doi:10.1186/s12864-023-09301-9)
Supplement: Supplementary file 5 — Additional file 5. [file 12864_2023_9301_MOESM5_ESM.pdf]

Site frequency spectrum (SFS) of each locus under positive selection were given:

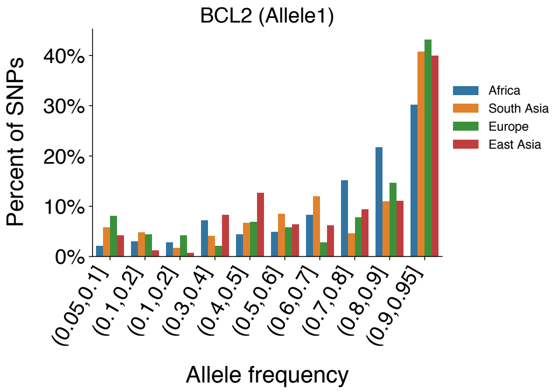

Figure S1 Site frequency spectrum of allele 1 of BCL2.

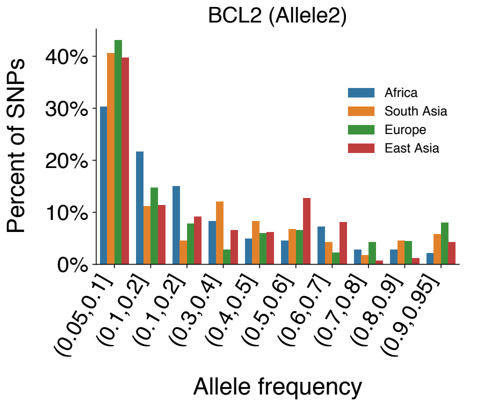

Figure S2 Site frequency spectrum of allele 2 of BCL2.

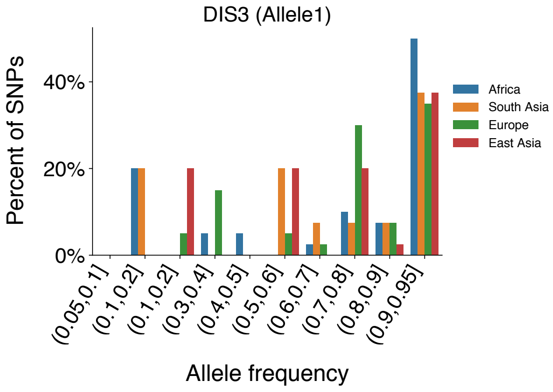

Figure S3 Site frequency spectrum of allele 1 of DIS3.

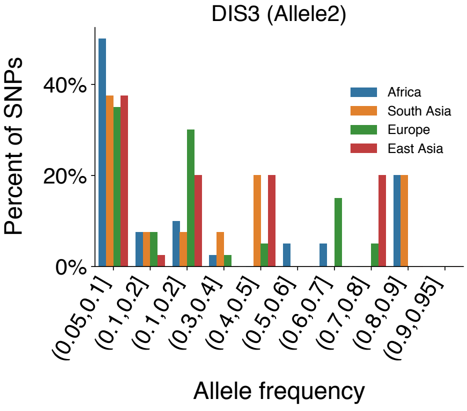

Figure S4 Site frequency spectrum of allele 2 of DIS3.

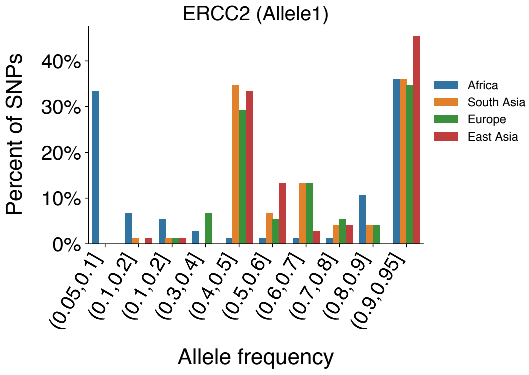

Figure S5 Site frequency spectrum of allele 1of ERCC2.

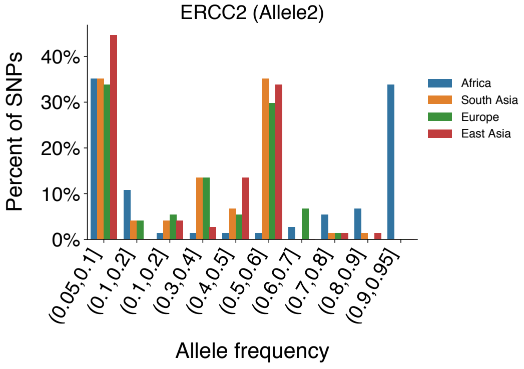

Figure S6 Site frequency spectrum of allele 2 of ERCC2.

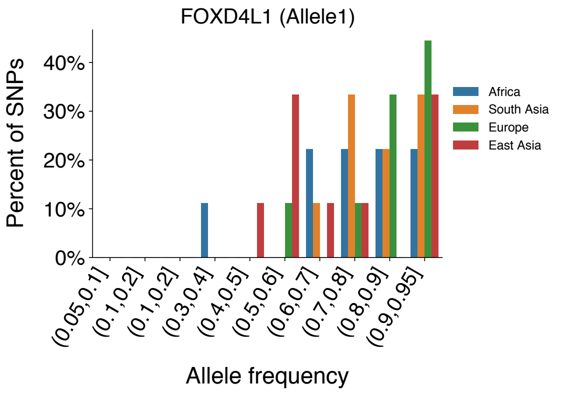

Figure S7 Site frequency spectrum of allele 1of FOXD4L1.

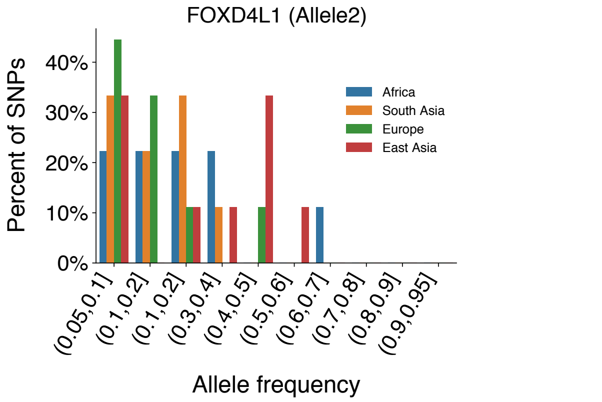

Figure S8 Site frequency spectrum of allele 2 of FOXD4L1.

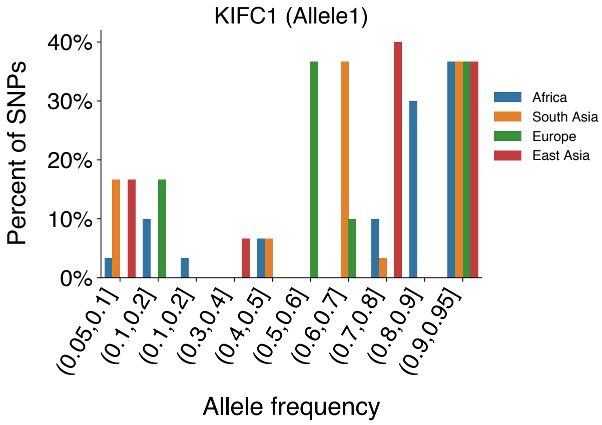

Figure S9 Site frequency spectrum of allele 1of KIFC1.

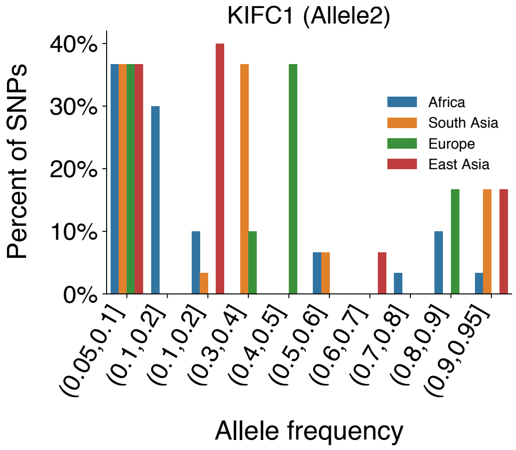

Figure S10 Site frequency spectrum of allele 2 of KIFC1.

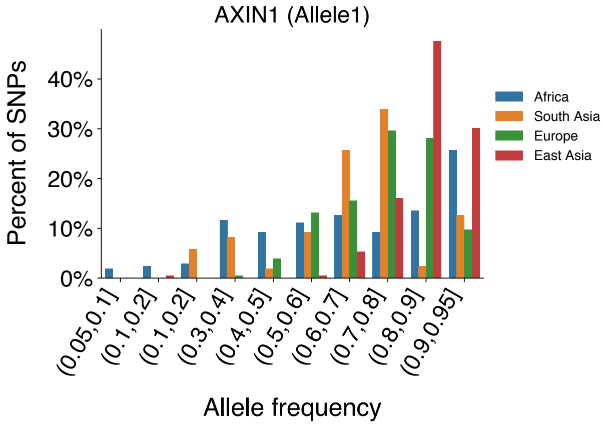

Figure S11 Site frequency spectrum of allele 1of AXIN1.

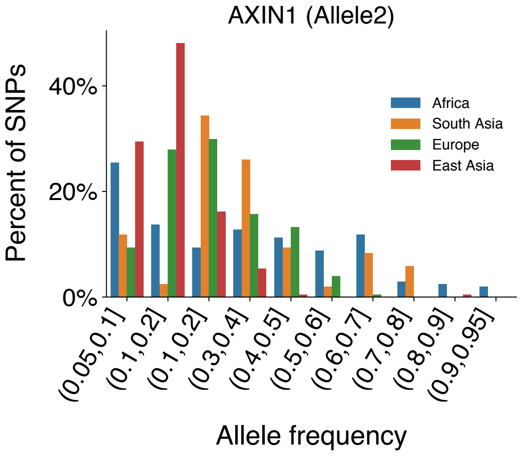

Figure S12 Site frequency spectrum of allele 2 of AXIN1.

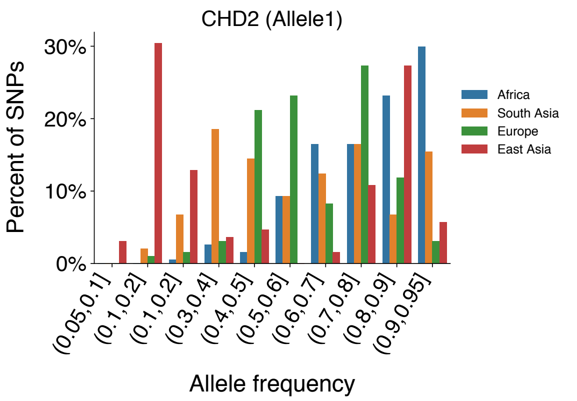

Figure S13 Site frequency spectrum of allele 1of CHD2.

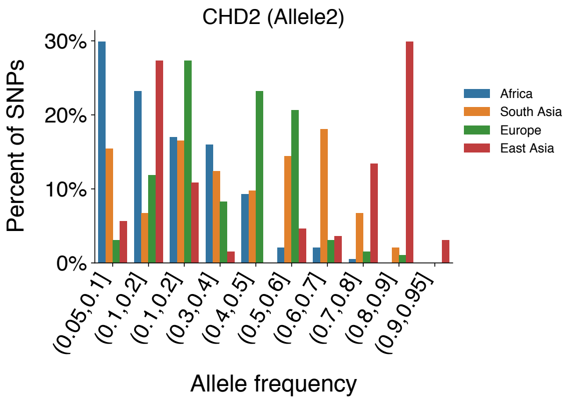

Figure S14 Site frequency spectrum of allele 2 of CHD2.

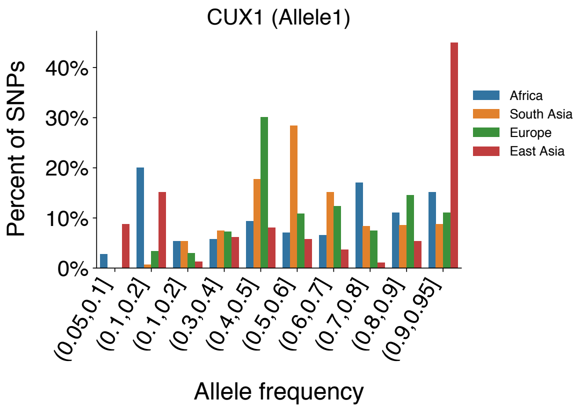

Figure S15 Site frequency spectrum of allele 1of CUX1.

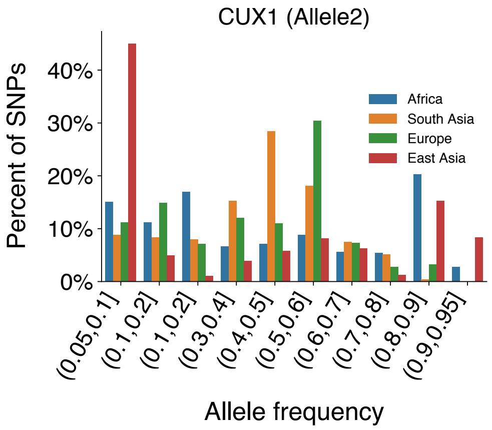

Figure S16 Site frequency spectrum of allele 2 of CUX1.

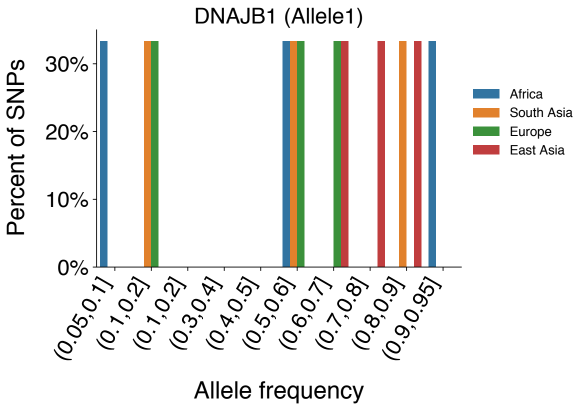

Figure S17 Site frequency spectrum of allele 1of DNAJB1.

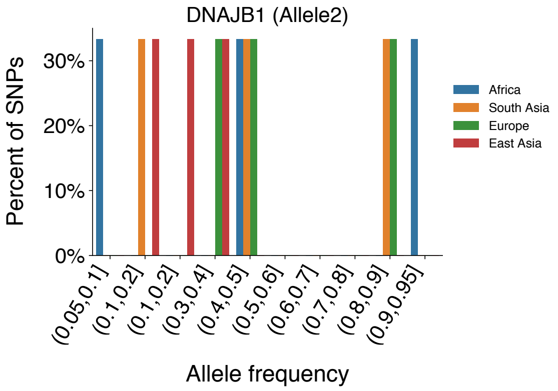

Figure S18 Site frequency spectrum of allele 2 of DNAJB1.

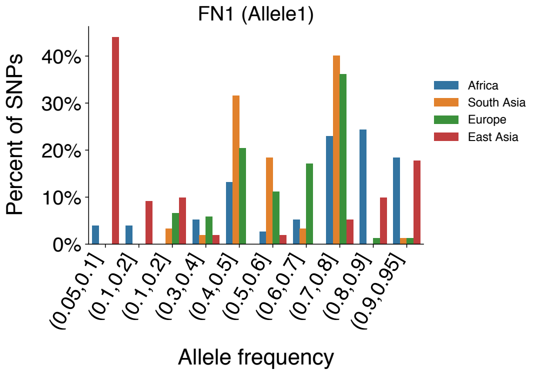

Figure S19 Site frequency spectrum of allele 1of FN1.

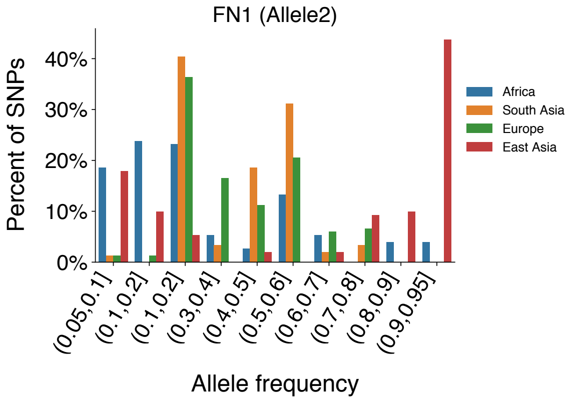

Figure S20 Site frequency spectrum of allele 2 of FN1.

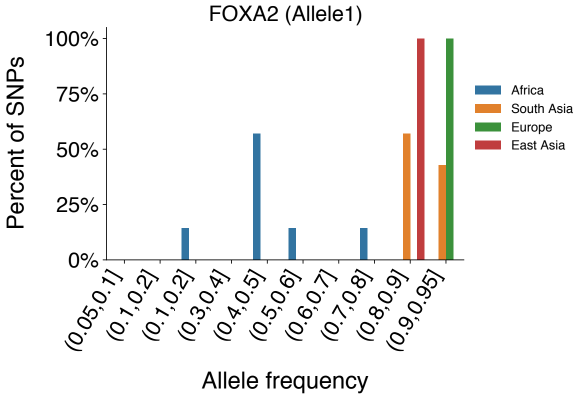

Figure S21 Site frequency spectrum of allele 1of FOXA2.

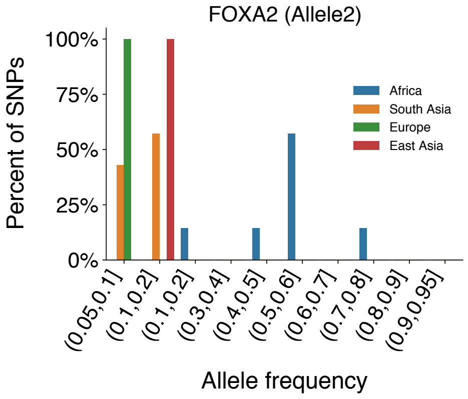

Figure S22 Site frequency spectrum of allele 2 of FOXA2.

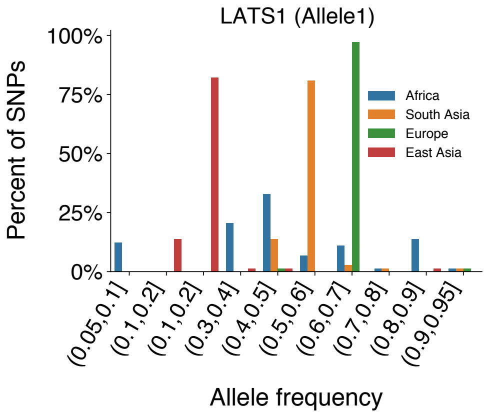

Figure S23 Site frequency spectrum of allele 1of LATS1.

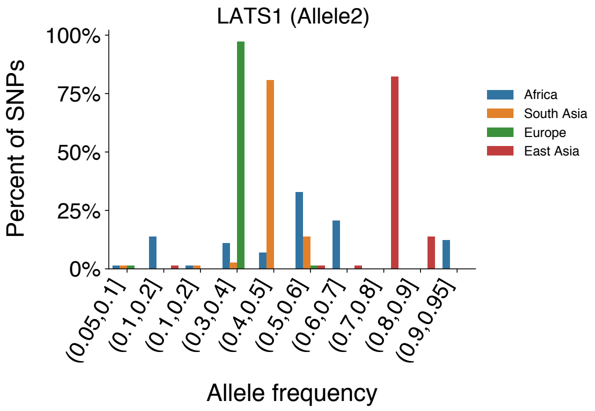

Figure S24 Site frequency spectrum of allele 2 of LATS1.

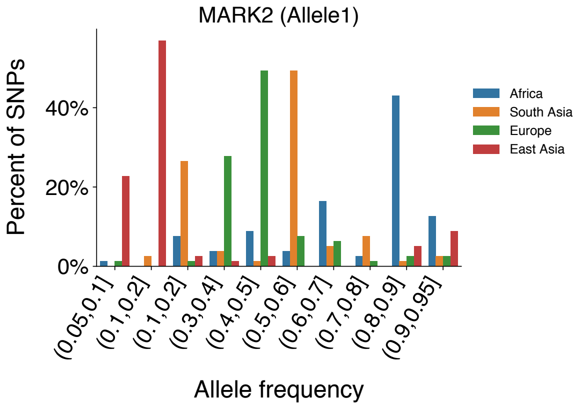

Figure S25 Site frequency spectrum of allele 1of MARK2.

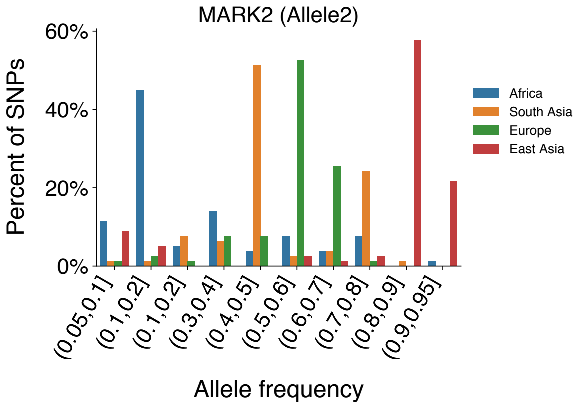

Figure S26 Site frequency spectrum of allele 2 of MARK2.

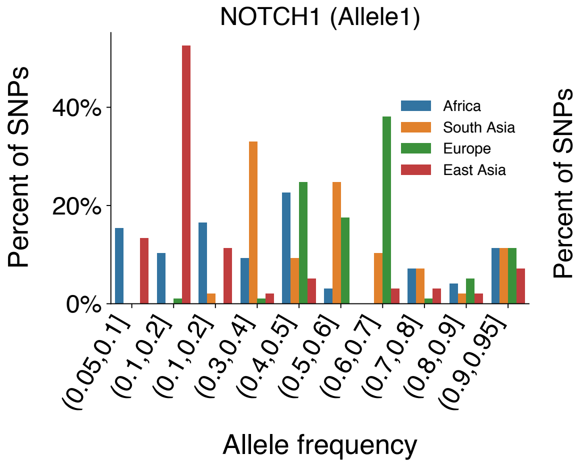

Figure S27 Site frequency spectrum of allele 1of NOTCH1.

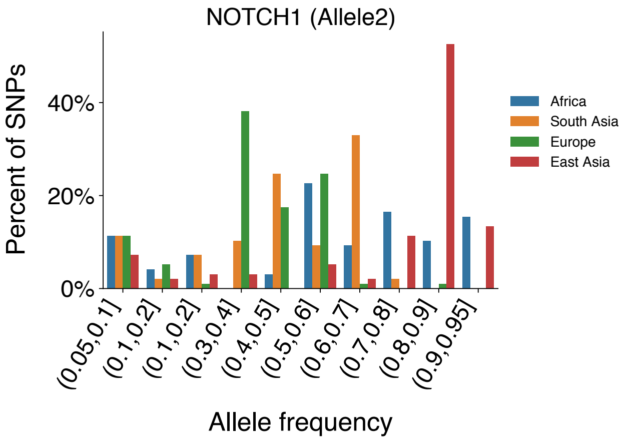

Figure S28 Site frequency spectrum of allele 2 of NOTCH1.

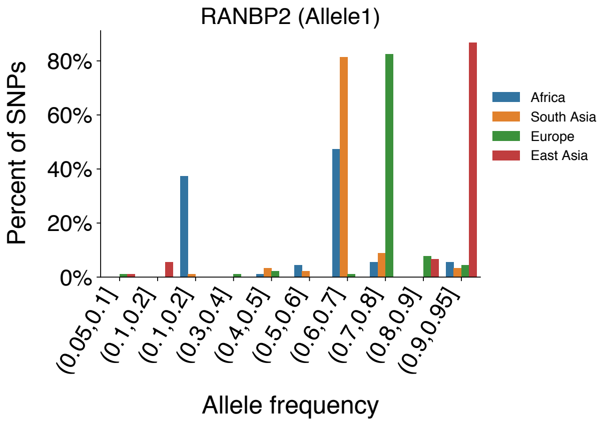

Figure S29 Site frequency spectrum of allele 1of RANBP2.

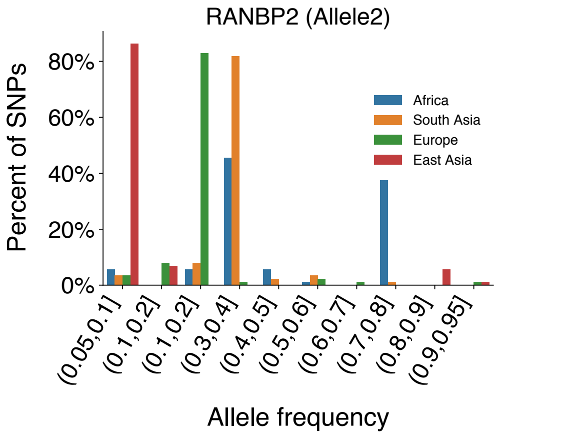

Figure S30 Site frequency spectrum of allele 2 of RANBP2.

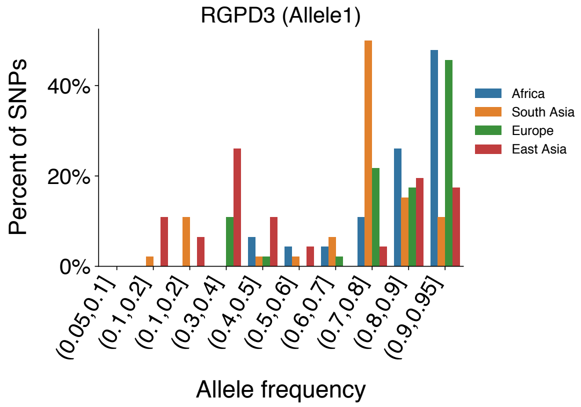

Figure S31 Site frequency spectrum of allele 1of RGPD3.

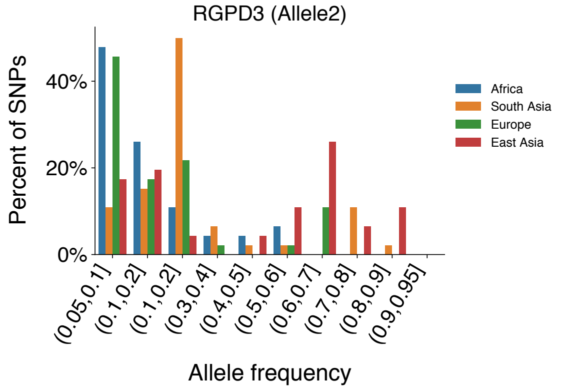

Figure S32 Site frequency spectrum of allele 2 of RGPD3.

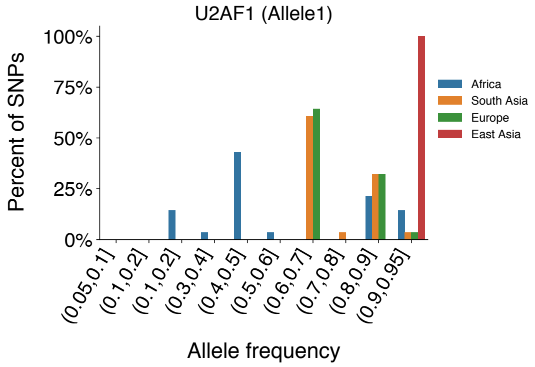

Figure S33 Site frequency spectrum of allele 1of U2AF1.

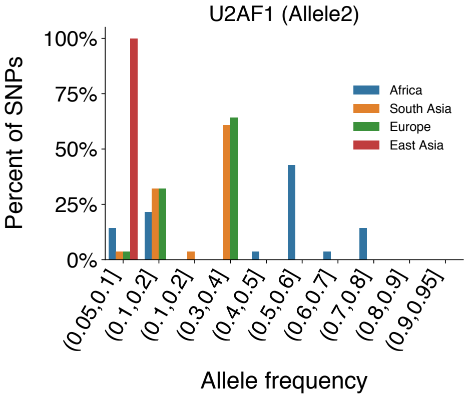

Figure S34 Site frequency spectrum of allele 2 of U2AF1.

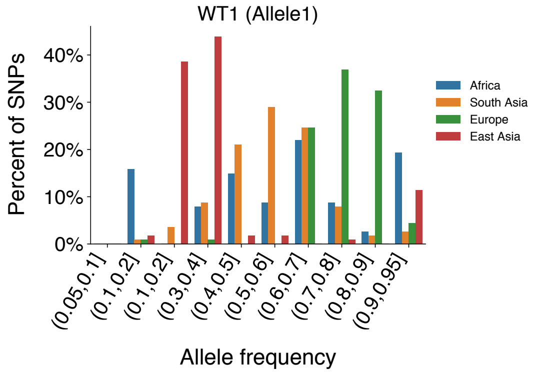

Figure S35 Site frequency spectrum of allele 1of WT1.

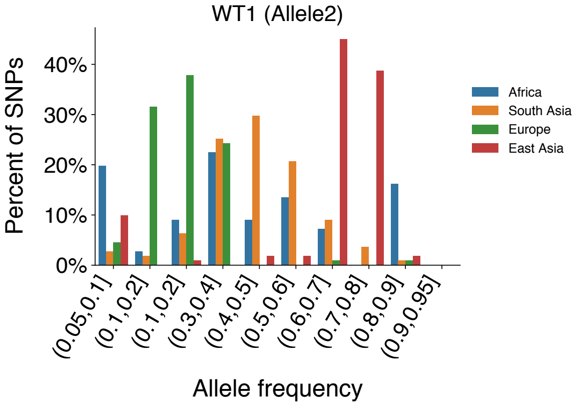

Figure S36 Site frequency spectrum of allele 2 of WT1.

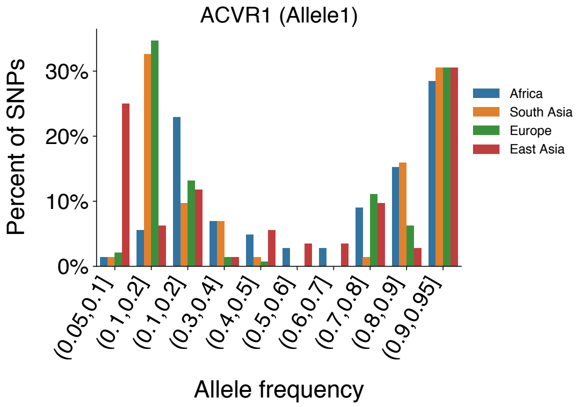

Figure S37 Site frequency spectrum of allele 1of ACVR1.

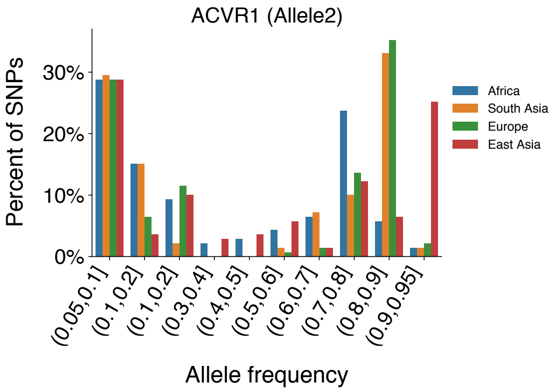

Figure S38 Site frequency spectrum of allele 2 of ACVR1.

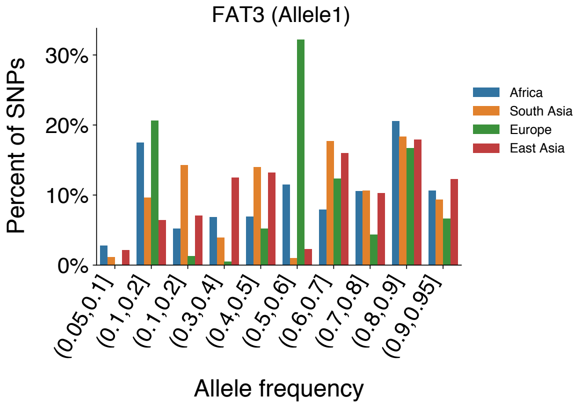

Figure S39 Site frequency spectrum of allele 1of FAT3.

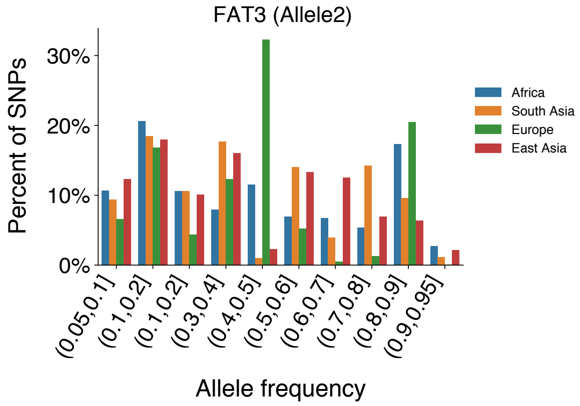

Figure S40 Site frequency spectrum of allele 2 of FAT3.

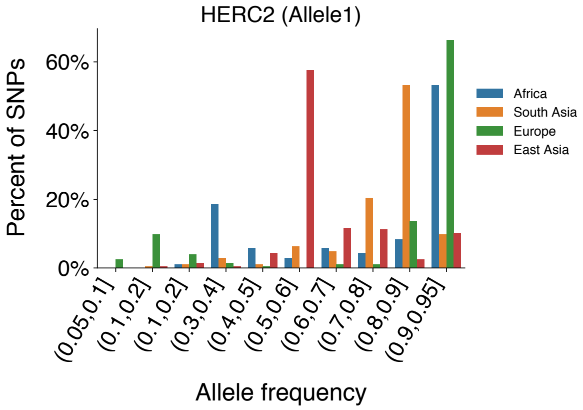

Figure S41 Site frequency spectrum of allele 1of HERC2.

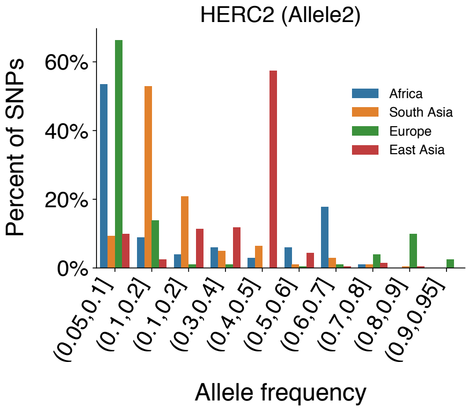

Figure S42 Site frequency spectrum of allele 2 of HERC2.

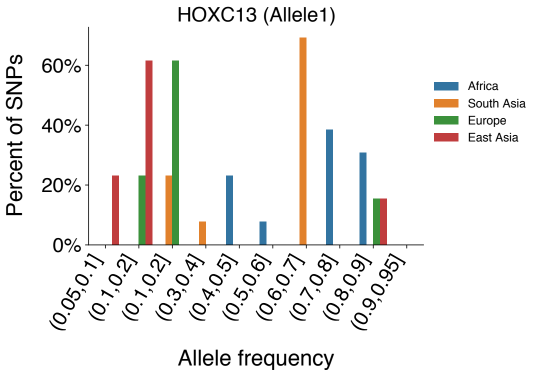

Figure S43 Site frequency spectrum of allele 1of HOXC13.

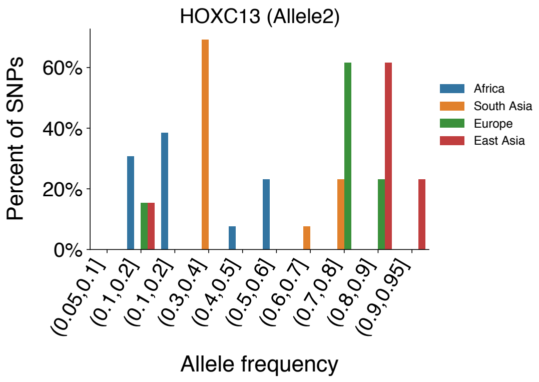

Figure S44 Site frequency spectrum of allele 2 of HOXC13.

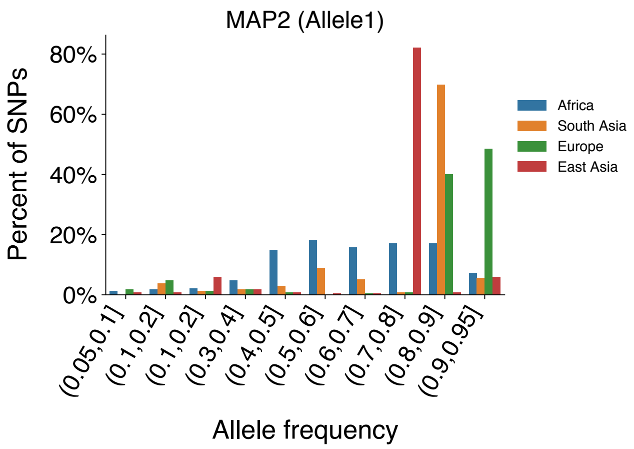

Figure S45 Site frequency spectrum of allele 1of MAP2.

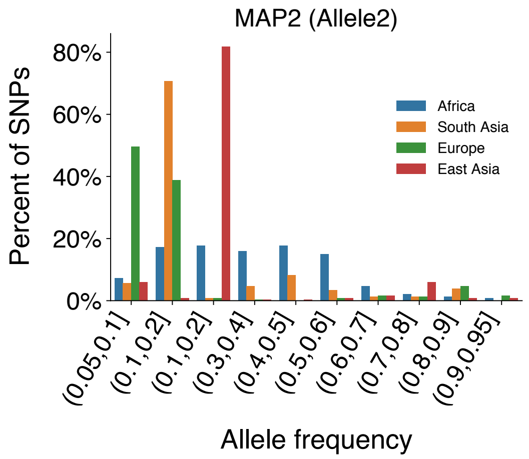

Figure S46 Site frequency spectrum of allele 2 of MAP2.

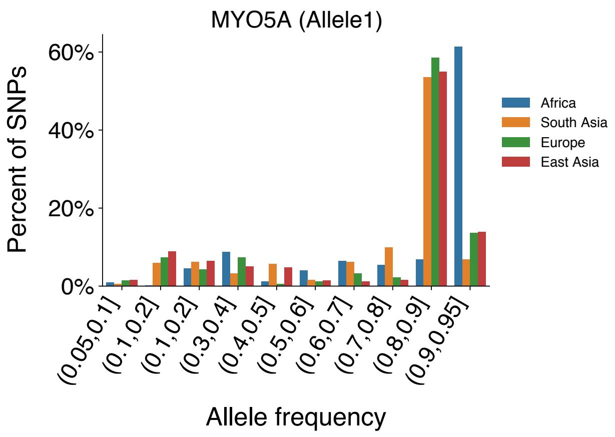

Figure S47 Site frequency spectrum of allele 1of MYO5A.

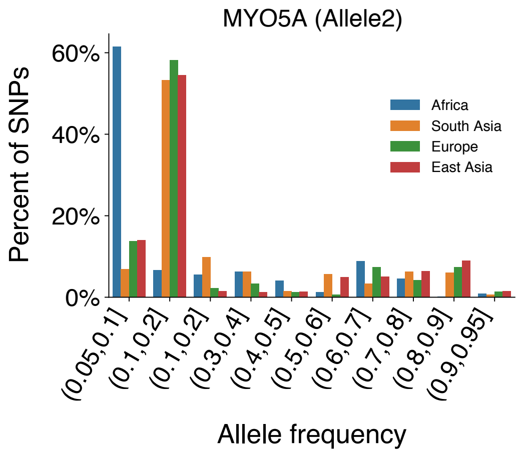

Figure S48 Site frequency spectrum of allele 2 of MYO5A.

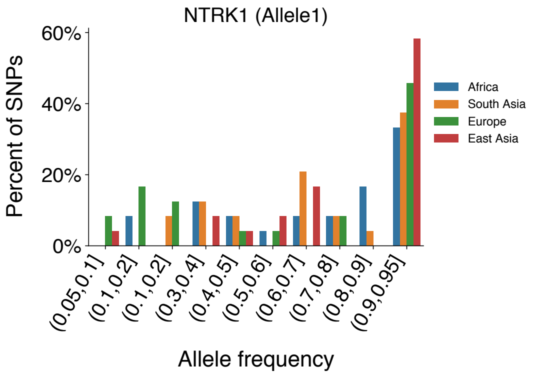

Figure S49 Site frequency spectrum of allele 1of NTRK1.

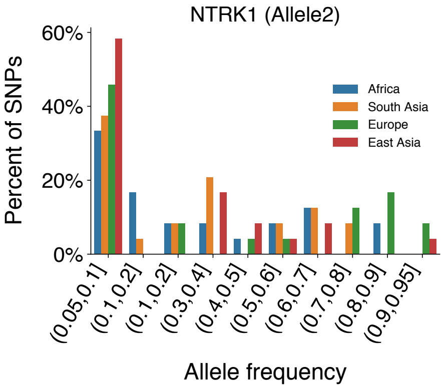

Figure S50 Site frequency spectrum of allele 2 of NTRK1.

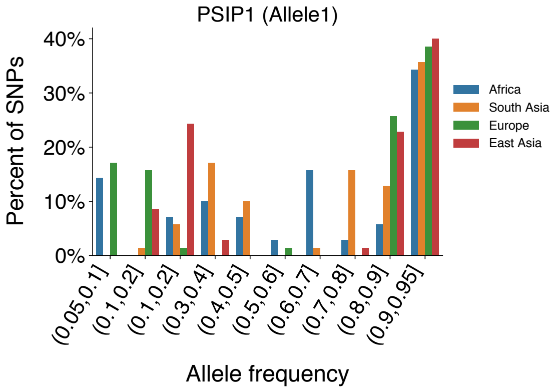

Figure S51 Site frequency spectrum of allele 1of PSIP1.

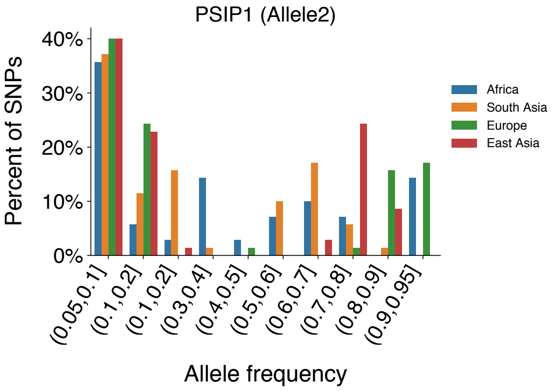

Figure S52 Site frequency spectrum of allele 2 of PSIP1.

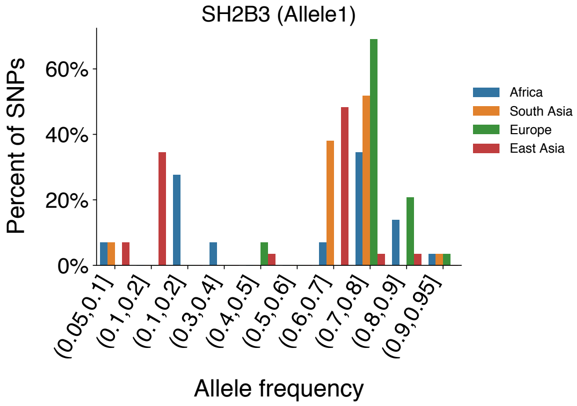

Figure S53 Site frequency spectrum of allele 1of SH2B3.

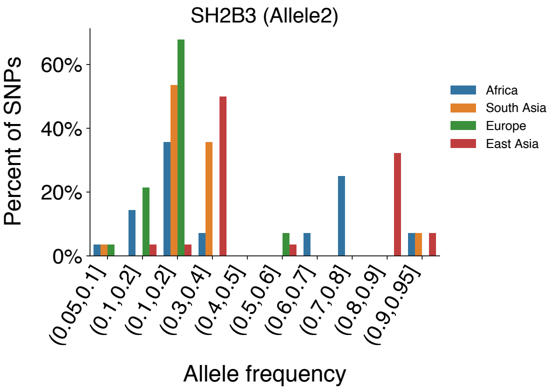

Figure S54 Site frequency spectrum of allele 2 of SH2B3.

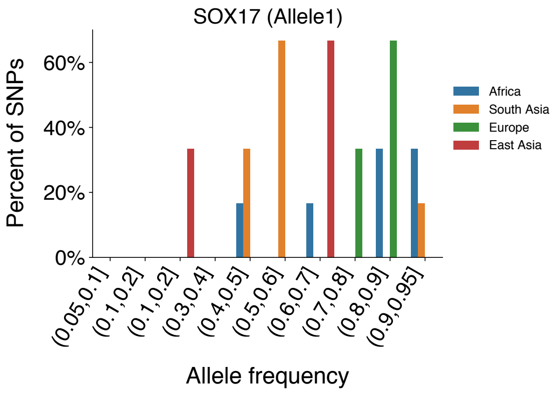

Figure S55 Site frequency spectrum of allele 1of SOX17.

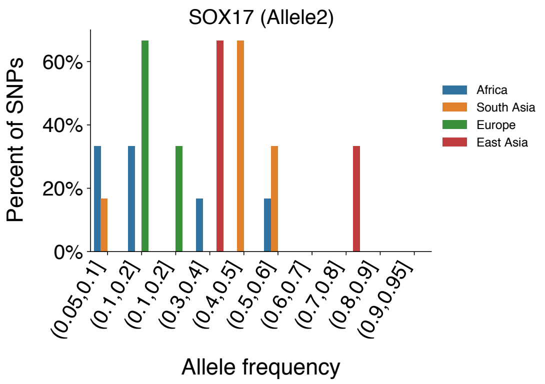

Figure S56 Site frequency spectrum of allele 2 of SOX17.

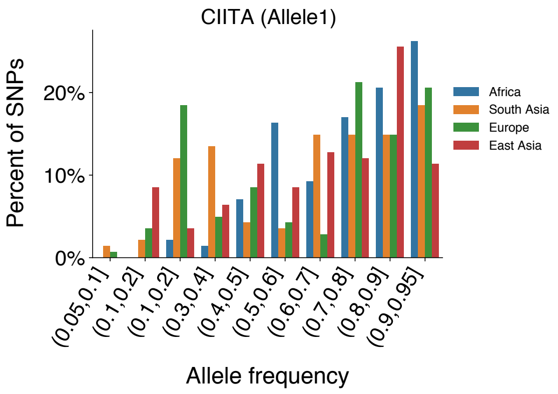

Figure S57 Site frequency spectrum of allele 1of CIITA.

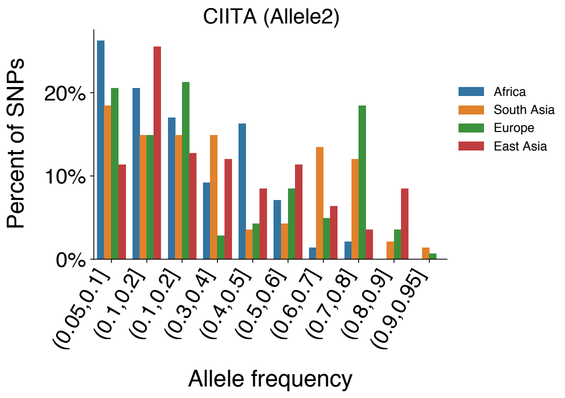

Figure S58 Site frequency spectrum of allele 2 of CIITA.

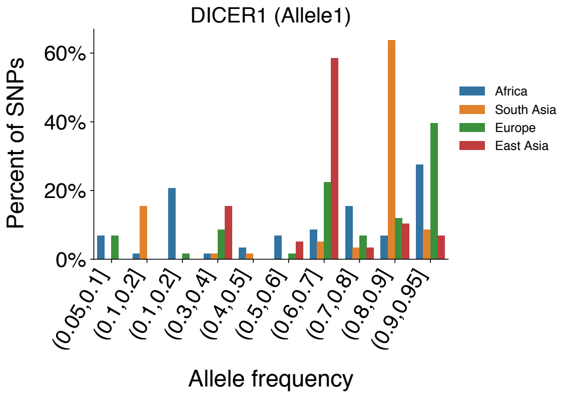

Figure S59 Site frequency spectrum of allele 1of DICER1.

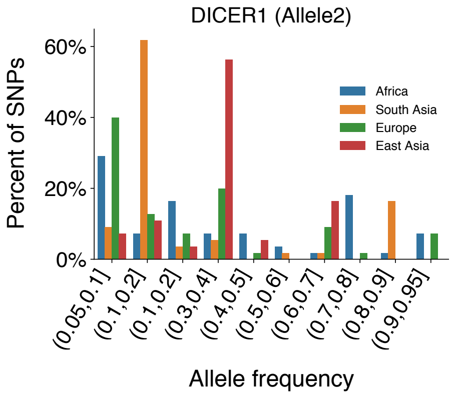

Figure S60 Site frequency spectrum of allele 2 of DICER1.

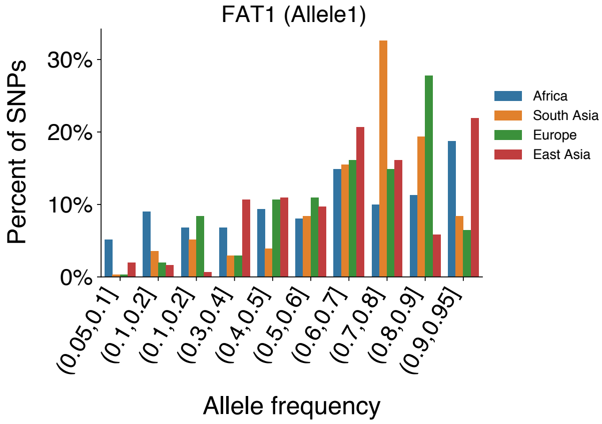

Figure S61 Site frequency spectrum of allele 1of FAT1.

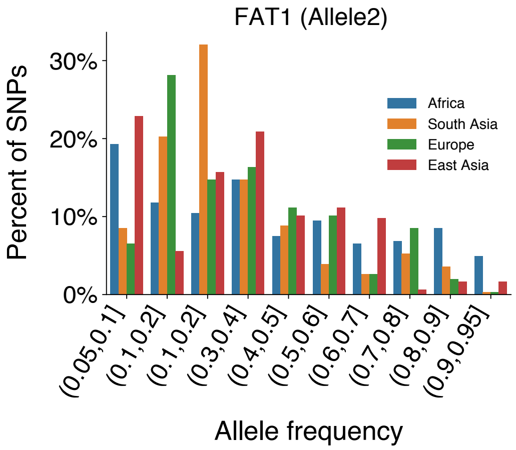

Figure S62 Site frequency spectrum of allele 2 of FAT1.

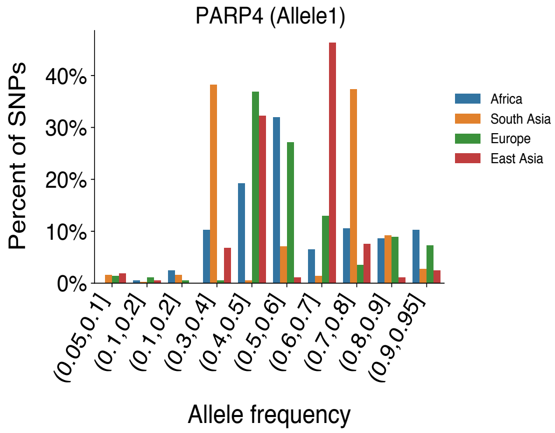

Figure S63 Site frequency spectrum of allele 1of PARP4.

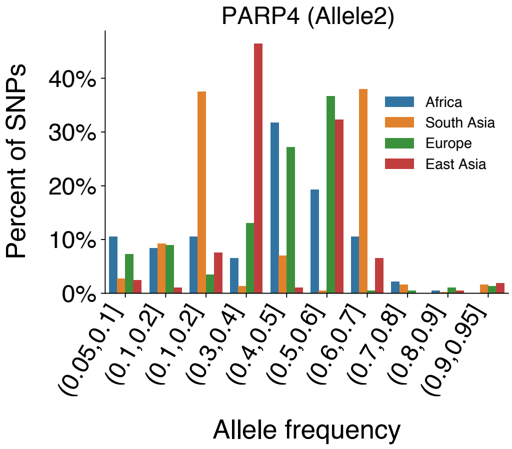

Figure S64 Site frequency spectrum of allele 2 of PARP4.

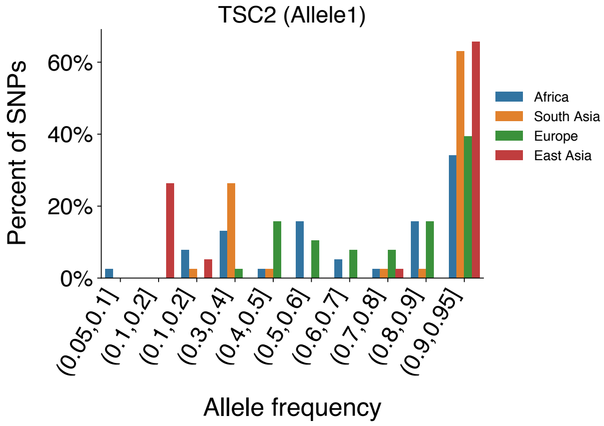

Figure S65 Site frequency spectrum of allele 1of TSC2.

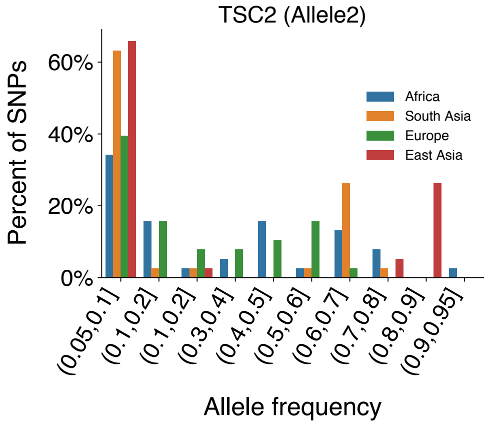

Figure S66 Site frequency spectrum of allele 2 of TSC2.

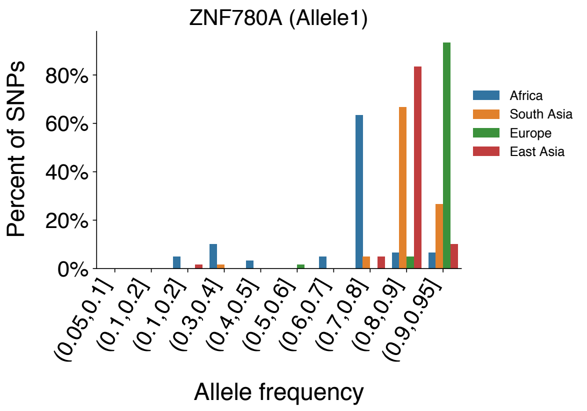

Figure S67 Site frequency spectrum of allele 1of ZNF780A.

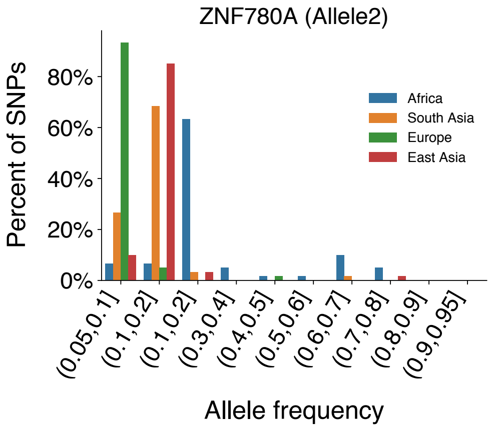

Figure S68 Site frequency spectrum of allele 2 of ZNF780A.

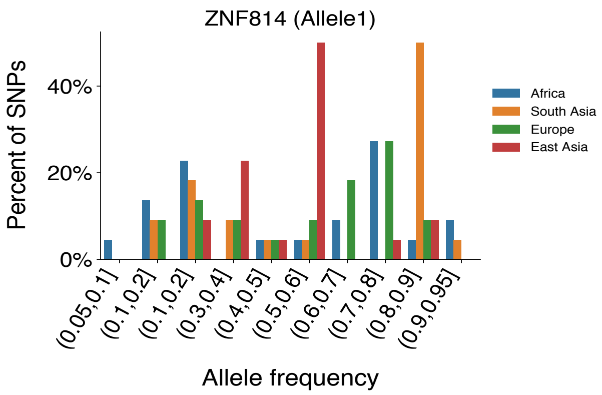

Figure S69 Site frequency spectrum of allele 1of ZNF814.

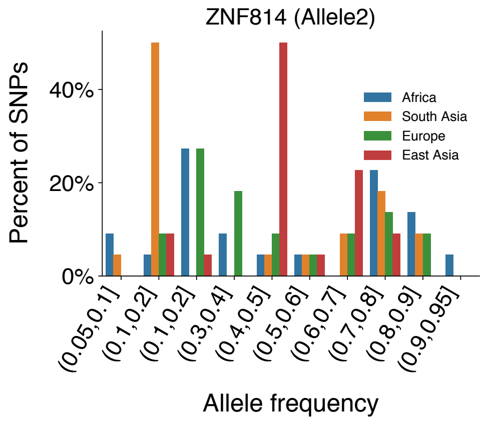

Figure S70 Site frequency spectrum of allele 2 of ZNF814.
